# Aging increases senescence, calcium signaling, and extracellular matrix deposition in human airway smooth muscle

**DOI:** 10.1371/journal.pone.0254710

**Published:** 2021-07-29

**Authors:** Sarah A. Wicher, Benjamin B. Roos, Jacob J. Teske, Yun Hua Fang, Christina Pabelick, Y. S. Prakash

**Affiliations:** 1 Department of Anesthesiology and Perioperative Medicine, Mayo Clinic, Rochester, MN, United States of America; 2 Department of Physiology and Biomedical Engineering, Mayo Clinic, Rochester, MN, United States of America; University of Debrecen, HUNGARY

## Abstract

Lung function declines as people age and their lungs become stiffer. With an increasing elderly population, understanding mechanisms that contribute to these structural and functional changes in the aging lung is important. Part of the aging process is characterized by thicker, more fibrotic airways, and senile emphysema caused by changes in lung parenchyma. There is also senescence, which occurs throughout the body with aging. Here, using human airway smooth muscle (ASM) cells from patients in different age groups, we explored senescence pathways and changes in intracellular calcium signaling and extracellular matrix (ECM) deposition to elucidate potential mechanisms by which aging leads to thicker and stiffer lungs. Senescent markers p21, γH2AX, and β-gal, and some senescence-associated secretory proteins (SASP) increased with aging, as shown by staining and biochemical analyses. Agonist-induced intracellular Ca^2+^ responses, measured using fura-2 loaded cells and fluorescence imaging, increased with age. However, biochemical analysis showed that expression of the following markers decreased with age: M_3_ muscarinic receptor, TRPC3, Orai1, STIM1, SERCA2, MMP2 and MMP9. In contrast, collagen III, and fibronectin deposition increased with age. These data show that senescence increases in the aging airways that is associated with a stiffer but surprisingly greater intracellular calcium signaling as a marker for contractility. ASM senescence may enhance fibrosis in a feed forward loop promoting remodeling and altered calcium storage and buffering.

## Introduction

Throughout life, the bronchial airways are exposed to environmental pollutants, allergens, and recurring respiratory infections resulting in cycles of injury, inflammation, and repair [1]. These insults contribute to changes in airway structure and function that manifest further as individuals age [2]. Indeed, lung function decreases starting around age 25 in females and age 30 in males with further acceleration after age 65 [1, 3–6]. Lung aging is characterized by thicker, more fibrotic airways [3, 7, 8]. Comparison of young and middle age lung tissue shows stiffness increases with age [9]. Such changes likely reflect increased quality or quantity of extracellular matrix (ECM) proteins such as collagens and fibronectin [7, 10]. ECM stiffness can also have relevance to airway contractility via impact on transmission of forces between cells and the ECM within bronchial airways [11], further impacted by inflammation or other insults [7, 12, 13]. Thus, age-related changes in the bronchial airways represent an important aspect of lung health and disease. There is currently limited to no data on aging-associated changes in ECM, particularly in the context of bronchial airways.

Airway tone and contraction are mediated by airway smooth muscle (ASM) involving bronchoconstrictors such as acetylcholine (ACh) or histamine that elevate intracellular calcium ([Ca^2+^]_i_) and contractility [12, 14, 15]. In ASM, regulation of [Ca^2+^]_i_ involves plasma membrane Ca^2+^ influx and sarcoplasmic reticulum (SR) Ca^2+^ release [16–18]. Aging has been shown to increase Ca^2+^ signaling and/or contractility in intestinal smooth muscle cells [19], fibroblasts [20], and vascular endothelial cells [21, 22]. However, characterization of Ca^2+^ signaling changes that occur in aging ASM is lacking.

It is now recognized that aging is associated with increased cellular senescence where accumulation of senescent cells also serves as a significant contributor to aging-associated changes in the structure and function of organs, overall leading to shortened lifespan [23–25]. Cellular senescence represents a state of replicative arrest induced by cellular stress including DNA damage, telomere shortening, and oxidative stress [26–28] that can be triggered by multiple intrinsic or environmental stimuli. Senescence is activated when accumulation of damage stimulates cyclin dependent kinases p21, and p16 to block cell cycle progression [24, 29]. Senescent cell effects are thought to occur via secretion of senescence associated secretory phenotype (SASP) proteins, which alter inflammation, production of extracellular matrix (ECM) and metabolism of surrounding cells and tissues: effects also observed in the lung [30–33] that could contribute altered lung structure and function. Natural aging occurs in the lungs [34–36] and thus senescence in the aging airways becomes relevant. Senescence has been shown to play an important role in patients with idiopathic pulmonary fibrosis (IPF) [31, 33, 37] where epithelial senescence SASP activates fibroblasts [38] which may also themselves be involved in senescence processes [31, 37, 39, 40]. Interestingly, senolytics have been shown to improve physical performance in patients with IPF [33, 41]. However, the impact of aging on senescence in ASM is currently not known.

In the present study, using non-diseased human ASM isolated from both male and female nonsmoking patients from 3 age groups as defined by the World Health Organization [42] (young (<45 years of age), middle age (45–65 years of age), and elderly (>65 years of age)) we tested the hypothesis that aging results in enhanced contractility and fibrosis, with increased cellular senescence that could contribute to the structural and functional changes seen with aging.

## Methods

### Isolation of human ASM cells

All procedures for obtaining human lung tissue were approved by the Mayo Clinic Institutional Review Board #16–009655 and considered minimal risk since samples were obtained incidental to patient surgery at the Mayo Clinic Rochester MN. Patients were informed and consented by research coordinators during their clinic visits prior to surgical decisions, and samples were obtained only from those patients who provided written or video/verbal consent followed by electronic signature for the use of their tissues and relevant medical records for research. Only adult patients were involved. Upon acquisition of tissues, relevant clinical data were recorded by the physicians involved in this study (Prakash, Pabelick), and all patient identifiers were deleted, and the samples given unique numbers to provide anonymization. The method for isolating ASM cells has been previously described [18, 43–45]. The 5^th^-6^th^ level bronchioles were isolated, the ASM layer was dissected, and tissue enzymatically dissociated using papain and collagenase with ovomuciod/albumin separation per manufacturer’s instructions (Worthington Biochemical, Lakewood, NJ). Isolated cells were then sub-cultured under standard conditions 37^○^C and 5% CO_2_ in DMEM/F12 (Gibco, Gaithersburg, MD) without phenol red and supplemented with 1% antibiotic/antimycotic (Gibco, Gaithersburg, MD) and 10% FBS (R&D Systems, Minneapolis, MN) until 80% confluent. Since high passages of subculture can potentially alter cellular phenotype, experiments were limited to subculture passages 1–5 to ensure maintenance of ASM phenotype. Accordingly, depending on tissue sample amount obtained from patients, it was not possible to always obtain sufficient numbers of cells to perform all protocols using any single patient sample. Where possible, particularly for protocols requiring smaller numbers of cells, statistical rigor was increased using greater numbers of patients. Media was changed every other day. Prior to experiments, cells were serum starved for 96h.

Patients defined as normal for this study had no prior documented or reported history of lung disease, and were clinically deemed to have otherwise normal lung function. For this study, samples from both male and female adult non-smokers were utilized, with ages ranging from 20–44 years for young, 51–64 years for middle age, and 65–83 years for elderly.

### Quantitative RT-PCR

Total RNA was extracted from cells and standard techniques used to synthesize and amplify cDNA using a Roche LightCycler LC480. RT-PCR was performed in triplicate per cDNA template. Ct values were normalized to S16, and fold change was calculated by the ΔΔCt method all data was normalized to an average of young gene expression. Primers used from Qiagen P21 (CDNK1A_1_SG QT00062090), p16 (CDNK2A_1_SG QT00089964), and p53 (p53_1_SG QT00060235). S16 forward (GCTTTCCTTTTCCGGTTGCG), S16 reverse (ACACGGATGTCTACACCAGC) were ordered individually from IDT, Iowa City, Iowa.

### ECM deposition

ASM were grown to confluence in black clear bottom 96 well plates and serum starved for 96 h. Following treatment per experimental protocol, cell number was quantified using a MTS Assay (Promega, Madison, WI), washed with PBS, and decellularized using 0.016 N NH_4_OH for 30 min. Cellular removal was confirmed visually ensuring that only ECM remained. ECM deposition was then measured using a semi-quantitative Li-Cor In-Cell western technique for collagen I (Abcam ab34710), collagen III (Abcam ab7778), and fibronectin (Abcam ab2413). ECM fluorescence intensity was normalized to cell number.

### Western blot analysis

Protein expression was measured using WES (ProteinSimple) with appropriate primary and secondary antibodies validated for this capillary based electrophoresis system. Protein expression was quantified using Compass for SW Software. Antibodies were used at a 1:50 dilution unless otherwise noted. P21 (Abcam ab9260), p53 (Cell signaling 9282), phosphor-p53 (Cell signaling 9284), MMP2 (Cell signaling 4022), MMP9 (cell signaling 2270), M3 Muscarinic Receptor (Abcam 126168), Histamine H1 Receptor (Novus NBP1-06039), Orai1 (Alomone ACC-060), SERCA1 (Alomone ACP-011), SERCA2 (Alomone ACP-012), TRPC3 (Alomone ACC-016) STIM1 abcam ab62031) and STIM2 (Novus NBP1-76790). Protein expression was normalized to GAPDH (Cell Signaling 1:200) and reported as fold change from the average values in the young group. Antibodies for p53, p21, P-p53, γH2aX, ki-67, collagen I, collagen III, fibronectin and GAPDH were previously validated by Parikh et al. and You et al. [30, 32]. Antibodies for MMP2 and MMP9 were also validated by Yang et al. [46]. TRPC3, Orai1 and STIM1 were validated by Abcejo et al. [47]. SERCA1, SERCA2, STIM2 and histamine H1 receptor antibody were validated using mouse brain lysate.

### Proliferation

Adult ASM cells were plated in a 12 well plate at a density of 8000 cells per well in DMEMF12 containing 1% FBS and 1% Antibiotic/Antimycotic. Cells were allowed to grow for 3 days, fixed with 4% paraformaldehyde, permeabilized, immunostained for Ki67 (Abcam ab9260, 1:200) using standard procedures (nuclei stained with DAPI) and imaged using fluorescence microscopy [30]. Positive cells were determined in each sample from 25 randomly selected fields at 10x magnification using a Bio-Tek Cytation5, analyzed using a visual threshold by Bio-Tek Gen5Image software, and expressed as percentage of total cell number (DAPI counterstain). All assays were done in triplicate, and at least 200 cells were counted.

#### Immunostaining

Adult ASM were grown in a 4 well chamber slide to 70% confluence, serum starved (DMEMF12, 1% Antibiotic/Antimycotic) for 4 days, fixed with 4% paraformaldehyde, permeabilized, immunostained for p-γH2A.X (Abcam ab11174, 1:200), or p21 (Abcam ab9260, 1:200) using standard procedures (nuclei counterstained with DAPI), and imaged using a Keyence BX-800 system [30]. Positive cells were determined from 10 randomly selected fields of view at 40x magnification, and analyzed using visual threshold by ImageJ software, with positive cells expressed as percent of total cell number. All assays were done in triplicate, and at least 200 cells were counted. Personnel performing the fluorescence imaging and analysis were blinded for the age of the individual from whom ASM samples were derived.

### Senescence-associated b-galactosidase (SA-β-gal) activity

Cells were plated at 8000 per well grown to 70% confluence, initially grown in 1% serum for 2 days, and serum starved for 2 more days. SA-β-Gal was detected by colorimetric assay (Cell Signaling). Microscopic image analysis of 25 10x fields per experiment were done using a BioTek Cytation5. Using Gen5 microplate reader software total cell number was quantified by DAPI staining, while SA-β-Gal positive cells were quantified using imaging threshold of 5000 above baseline [32]. Personnel performing the SA-β-Gal imaging and analysis were blinded for the age of the individual from whom ASM samples were derived.

### Ca^2+^ imaging

To measure [Ca^2+^]_i_, ASM cells serum starved for 96h were loaded with 5 μM fura-2-AM (Invitrogen, Carlsbad, CA) for 30 min at room temperature and washed for 30 min in HBSS and visualized with a fluorescent imaging system (Nikon Eclipse Ti; standard fura-2 filters; Nikon Elements;). Cells were perfused with HBSS [2.5 mM Ca^2+^or 0 mM Ca, room temperature (23^○^C)], and a baseline fluorescence was established. [Ca^2+^]_i_ responses of 10–15 cells per chamber were obtained, with 2 individual software defined regions of interest per cell. Fura-2-AM loaded cells were alternately excited at 340 and 380 nm with a lambda 10–2 filter changer. Fluorescent emissions were measured separately for each wavelength with a 510 barrier filter at 1 sec intervals. Images were acquired with an Andor iXon ultra digital camera. Previously described calibration procedures were used to quantify [Ca^2+^]_i_ from fura-2-AM fluorescence levels [48, 49]. Cells were stimulated with contractile agonists histamine (10 μM) or ACh (10 μM). Following agonist stimulation, amplitudes of [Ca^2+^]_i_ were calculated as the difference between peak Ca^2+^ and baseline Ca^2+^ levels prior to agonist stimulation.

#### Store operated Ca^2+^ influx

SOCE was measured using previously described techniques [16, 17, 49]. Briefly, SR Ca^2+^ was passively depleted by 10 μM cyclopiazonic acid (CPA) for 5 min in the absence of extracellular Ca^2+^ (0mM Ca^2+^ HBSS containing 1 μM nifedipine, and 10 mM KCl), after which 2.5 mM extracellular Ca^2+^ was rapidly re-introduced in the continued presence of nifedipine, KCl, and CPA, and the observed [Ca^2+^]_i_ response measured.

### Statistical analysis

All data were obtained from at least 5 patients per age group, and protocols were repeated three times where relevant. Statistical analysis was performed using GraphPad Prism version 8.0.0. Statistical differences between experimental groups were analyzed using Student’s t-test or 1-way ANOVA followed by Dunnet or Tukey’s Post-hoc test for multiple comparisons where appropriate. Statistical significance was established at p≤0.05. Data shown as mean ± SEM.

## Results

### Senescence in aging ASM

**Fig 1:**
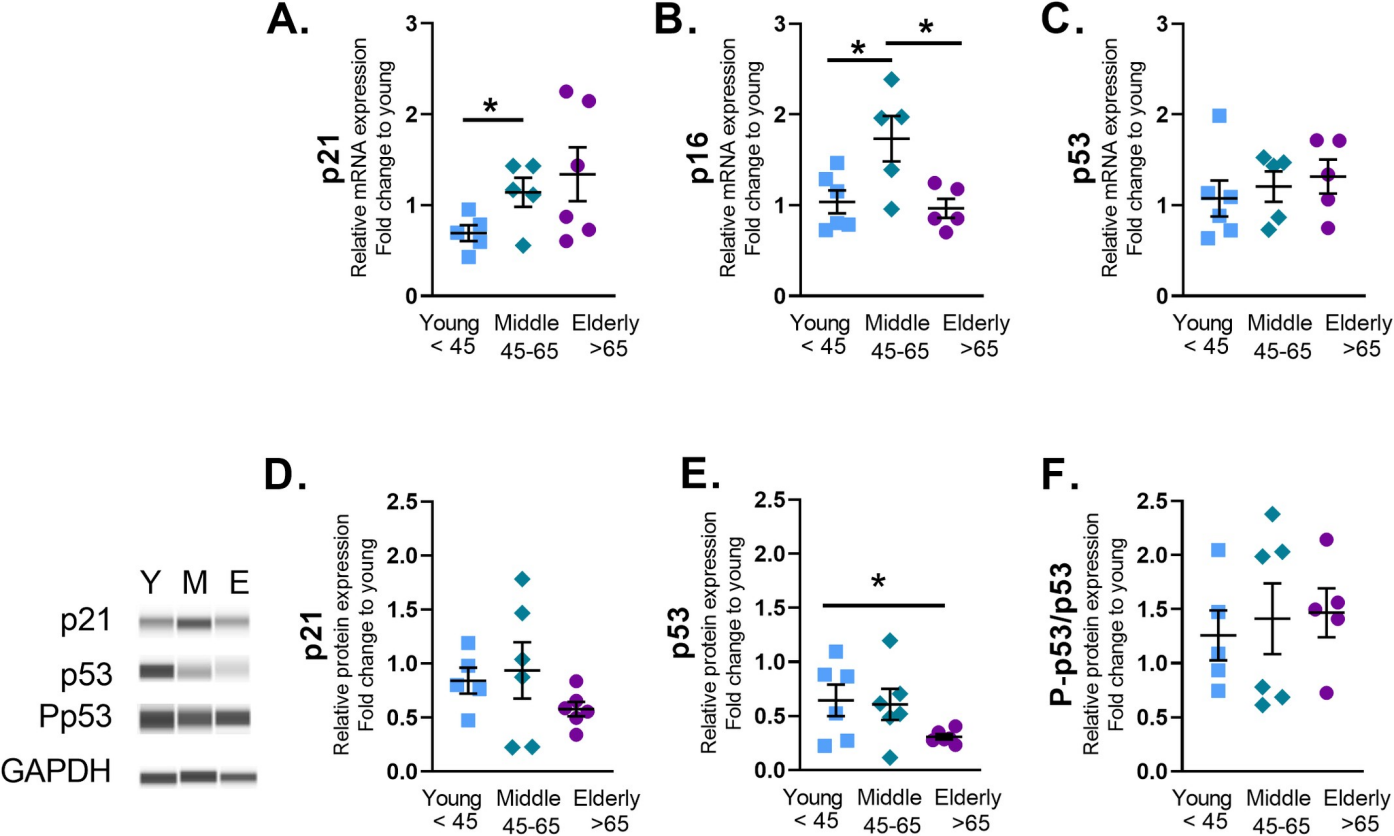
Aging increases senescence marker gene expression in human Airway Smooth Muscle (ASM) cells. mRNA was isolated from human ASM and p21, p16, and p53 gene expression was assessed by quantitative PCR. Compared to the young, p21 gene expression was increased in middle aged ASM cells (A). Senescent marker p16 was increased in middle aged ASM (B). Protein analysis for p21 (D), p53 (E) or p-p53 (F) normalized to GAPDH showed consistent changes for p53. Data are shown as mean ± SEM from *N* = 5–6 samples. Fold change was quantified against the average value of the samples from young individuals. * indicates significance *P* ≤ 0.05.

To quantify changes in senescence with age, we measured senescent marker gene expression in ASM cells from healthy young <45, middle age 45–65, and elderly >65 years humans. Real-time quantitative PCR analysis of cell cycle checkpoint genes demonstrated elevated mRNA for cell cycle arrest protein p21 in middle age ASM (Fig 1A). P16 gene expression was increased in middle aged ASM (Fig 1B), while p53 gene expression was not changed with age (Fig 1C). The range of Ct values measured for gene expression analysis are listed by age in S1 Table.

**Fig 2:**
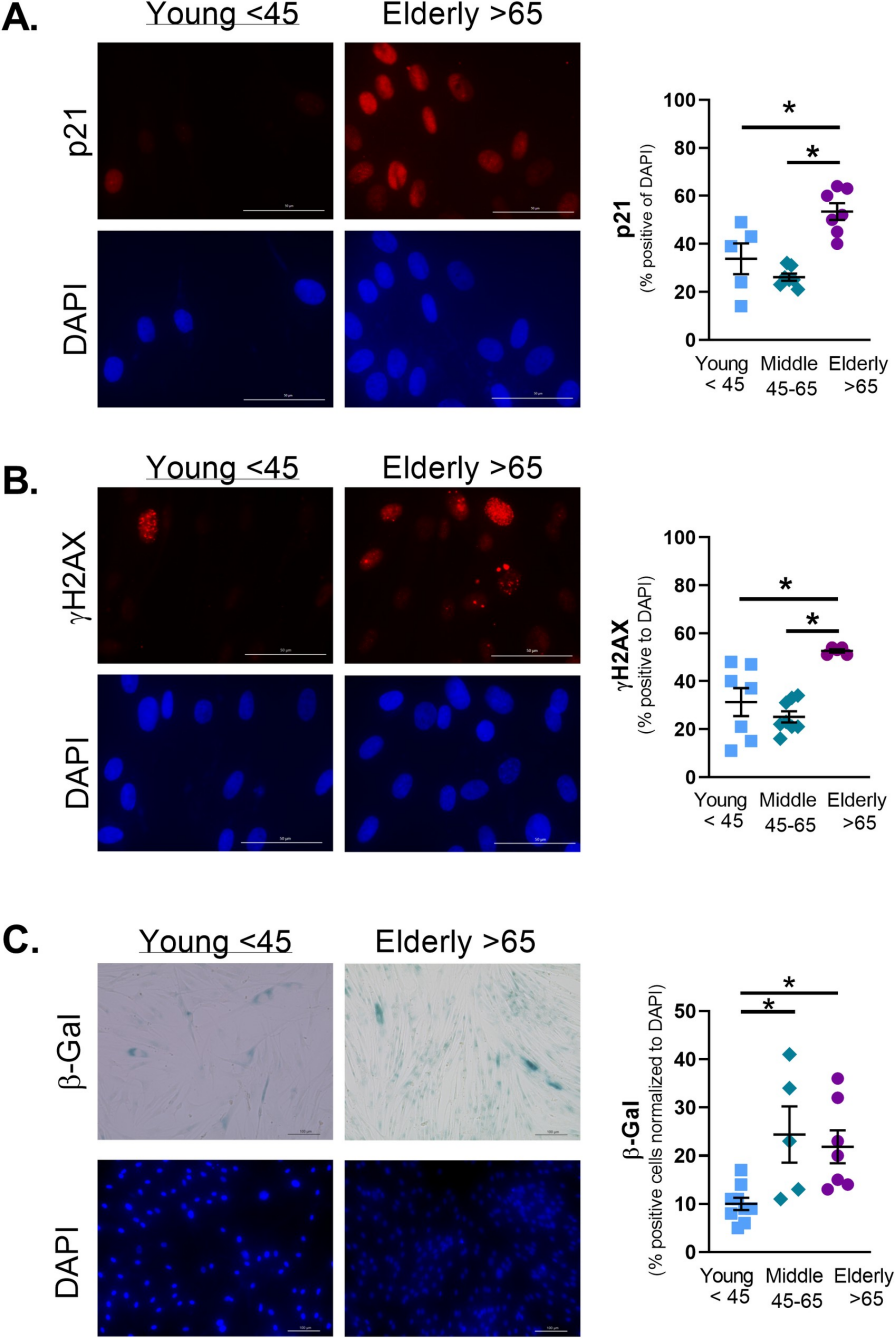
Aging increases senescence markers in ASM. Immunofluorescence staining for p21 a marker of cell cycle arrest (A) showed increased levels in elderly ASM. γH2AX, a marker for DNA damage, increased in elderly ASM (B). Staining for senescence-associated beta galactosidase (β-Gal) increased in both middle age and elderly ASM cells (C). Data shown as mean ± SEM from *n* = 5–7 patients. * indicates significantly different p≤0.05.

**Fig 3:**
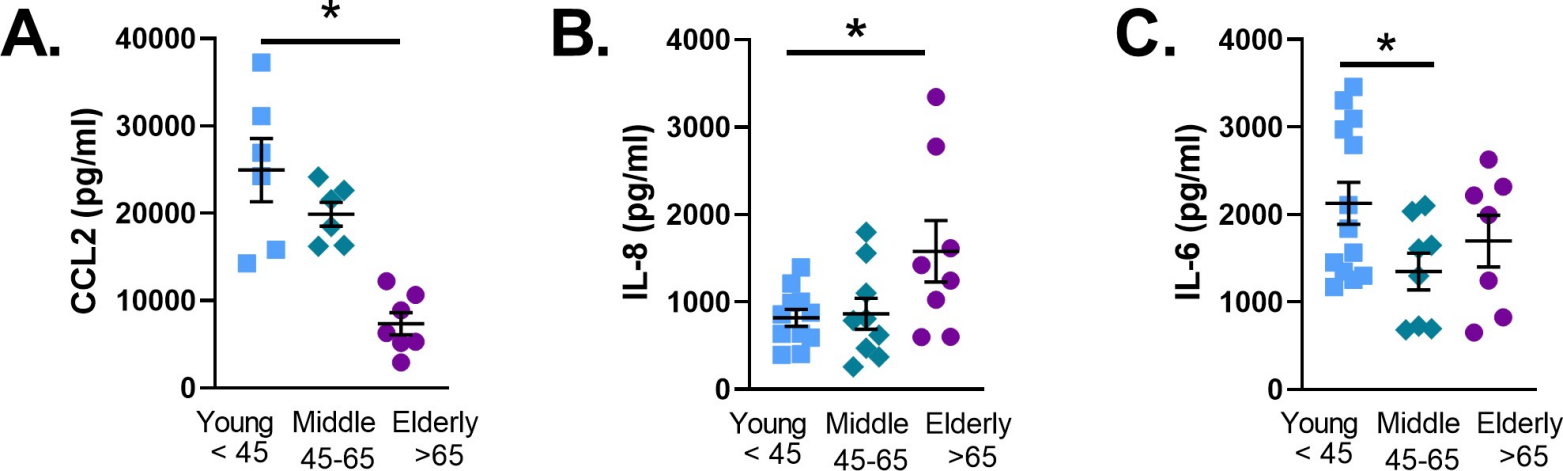
SASP release from ASM changes with age. Media were collected from ASM grown to confluence and serum starved for 4 days. Cytokine concentrations were measured via ELISA. CCL2 secretion was decreased in elderly ASM (A). IL-8 secretion increased in elderly ASM (B). IL-6 release decreased in middle aged ASM cells (C). Data shown as mean ± SEM from *n* = 7–9 patients. *indicates significant difference *P*≤0.05.

Protein analysis showed no change in p21 expression (Fig 1D), or P-p53 (Ser15) (Fig 1F). However, p53 protein expression was significantly decreased in the elderly (Fig 1E). Immunofluorescence staining of cell cycle arrest marker p21 (Fig 2A) and DNA damage marker Phospho-γH2AX (Fig 2B) showed significant increase in elderly ASM. SA-β-Gal is commonly used to identify senescent cells [32]. The percentage of SA-β-Gal positive cells increased in middle age ASM and remained elevated in elderly ASM (Fig 2C). The increase in senescence cells was accompanied by changes in SASP secretion by ASM. Elderly ASM release of CCL2 decreased when compared to both young and middle age cells (Fig 3A). In contrast, IL-8 secretion increased in elderly ASM (Fig 3B) while IL-6 secretion from middle age ASM decreased. In contrast, elderly ASM IL-6 secretion was similar to that of young ASM and thus relatively increased compared to middle age (Fig 3C).

### ASM proliferation and ECM deposition in aging

**Fig 4:**
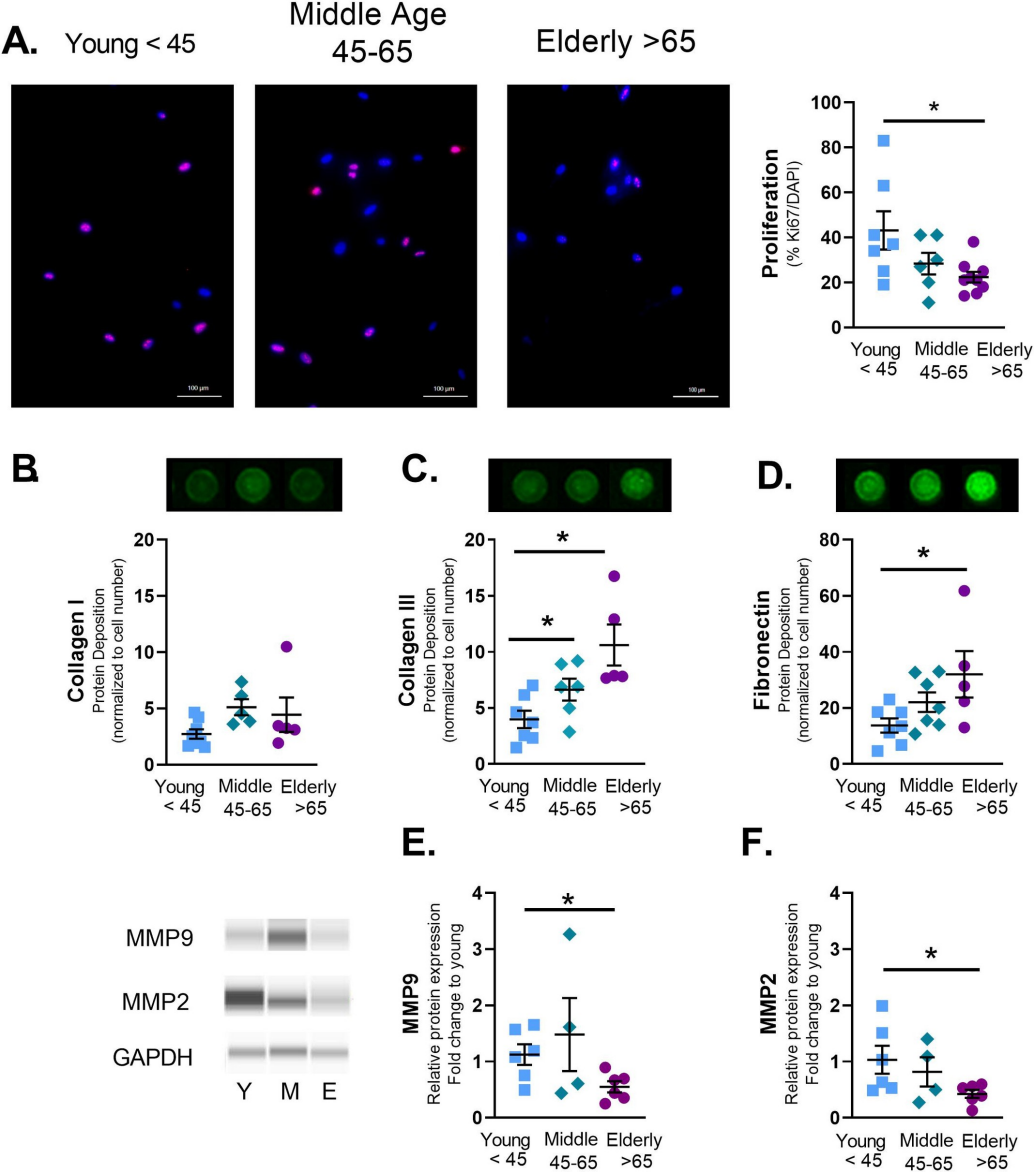
Aging alters ASM proliferation and extracellular matrix (ECM) deposition. To measure proliferation cells were plated in 1% serum and allowed to grow for 3 days. Cells were then stained for Ki67 and positive cells relative to total counts (DAPI) were measured. Proliferation decreased in elderly ASM cells (A). A modified Li-Cor In-Cell Western technique (semiquantitative immunofluorescence) was used to quantify deposition of ECM proteins by ASM grown to confluence and serum starved for 4 days. Aging increased deposition of collagen III (C), and fibronectin (D). Immunoblots showed aging-associated decrease in matrix modifying proteins MMP9 (E) and MMP2 (F). Data shown as mean ± SEM from *n* = 4–7 patients. * indicates significant difference *p≤0*.*05*.

Lung aging is associated with an increase in stiffness and airway thickening [3, 7, 8]. To understand how ASM cells contribute to increased stiffness with age, cell proliferation and ECM deposition were measured. Aging decreased proliferation of adult ASM cells measured by changes in Ki67 staining (Fig 4A). While collagen I showed no changes with aging (Fig 4B), collagen III (Fig 4C) and fibronectin (Fig 4D) deposition were increased with age. Furthermore, the ECM modifiers matrix metalloprotease-2 and -9 (MMP2 and 9) were decreased in elderly ASM (Fig 4E and 4F).

### ACh-induced [Ca^2+^]_i_ responses and aging

**Fig 5:**
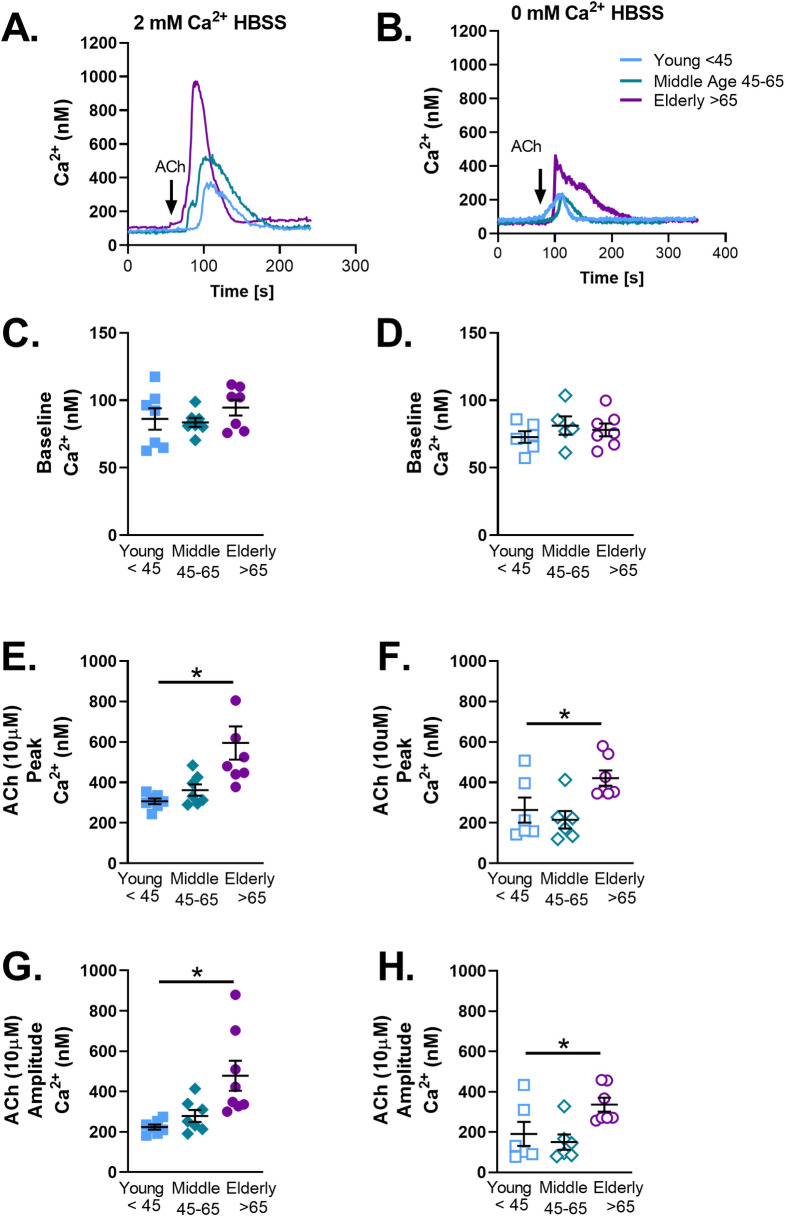
Acetylcholine-induced [Ca^2+^]_i_ in aging human ASM cells. Representative tracings in young <45, middle age 45–65, and elderly>65 human ASM (A&B). ACh (10μM, black arrow) was applied to Fura 2 AM loaded cells in 2 mM Ca^2+^ HBSS. Baseline intracellular calcium was not changed (C). Peak (E) and amplitude (G) of [Ca^2+^]_i_ was increased in elderly ASM. To measure changes in [Ca^2+^]_i_ release following contractile agonist stimulation independent of Ca^2+^ influx ACh (10μM) was applied to Fura 2 AM loaded cells in 0 mM Ca^2+^ HBSS (B). Baseline [Ca^2+^]_i_ was not changed (D). Peak (F) and amplitude (H) of [Ca^2+^]_i_ increased in elderly ASM. Data represents mean ± SEM from n of 6–7 patients. * indicates significant effect (p≤0.05).

ASM cells from young <45, middle age 45–65, and elderly >65 year old individuals were loaded with fura-2-AM and exposed to 10 μM ACh. Exposure to ACh resulted in the characteristic pattern of [Ca^2+^]_i_ peak followed by a return to baseline (Fig 5A). This pattern was maintained in the presence of 0 mM Ca^2+^ HBSS although the peak [Ca^2+^]_i_ response was significantly decreased (Fig 5B). Baseline [Ca^2+^]_i_ did not change with age in the presence of 2 mM Ca^2+^ (Fig 5C) or in zero extracellular Ca^2+^ (Fig 5D), while peak Ca^2+^ (Fig 5E and 5F) and amplitude (calculated as peak [Ca^2+^]_i_−baseline [Ca^2+^]_i_, (Fig 5G and 5H) responses significantly increased in elderly ASM. Interestingly, elderly ASM was more sensitive and demonstrated a pattern of spontaneous [Ca^2+^]_i_ release/waves in comparison to young ASM. This only occurred in the presence of 2 mM Ca^2+^ HBSS.

### Histamine-induced [Ca^2+^]_i_ responses and aging

**Fig 6:**
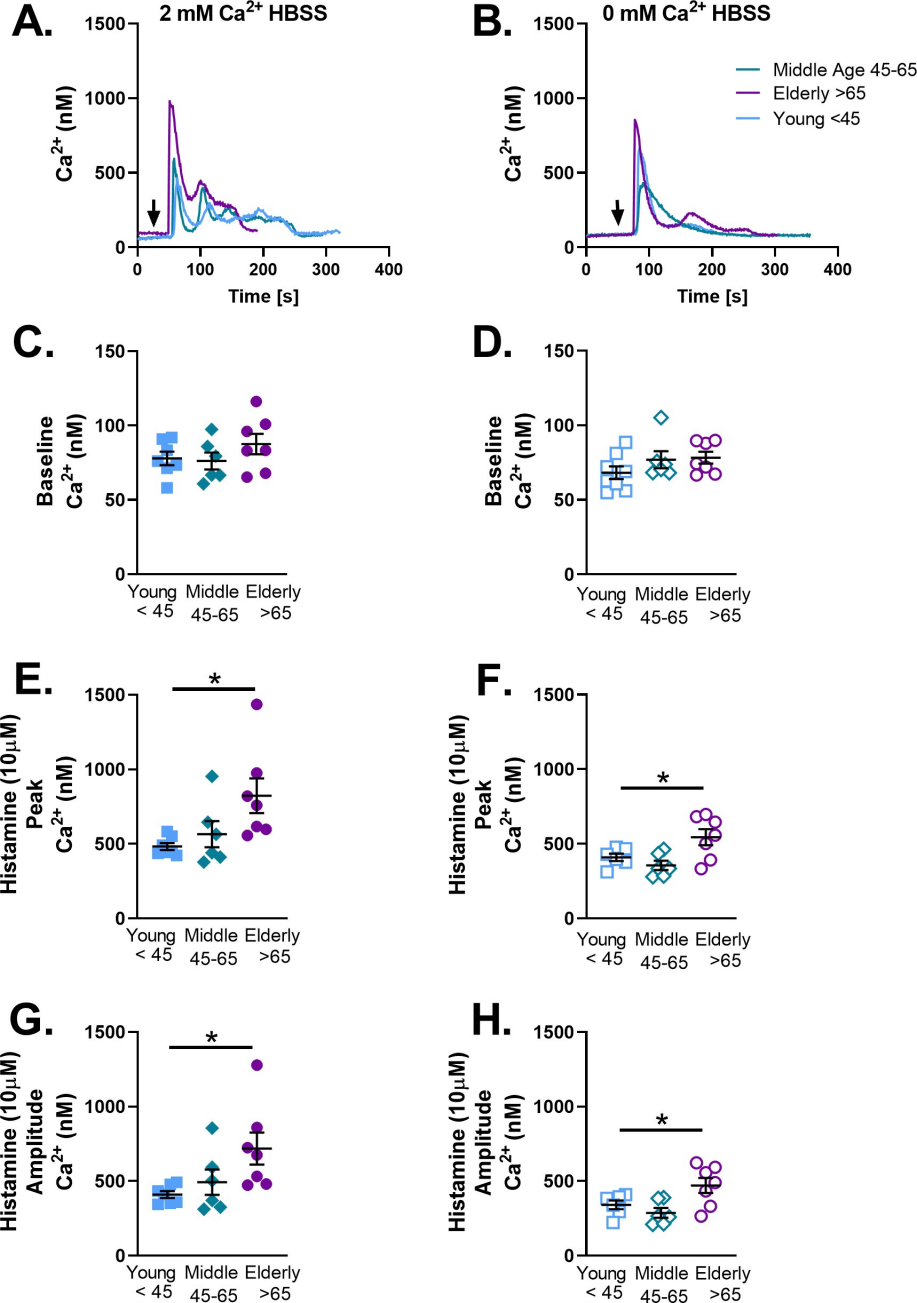
Histamine induced [Ca^2+^]_i_ increased in elderly human ASM. Representative tracings in young <45, middle age 45–65, and elderly>65 human ASM (A). Histamine (10μM, black arrow) was applied to Fura 2 AM loaded cells in 2 mM Ca^2+^ HBSS. Baseline [Ca^2+^]_i_ was not changed with age(C). After histamine peak (E) and amplitude (G) of [Ca^2+^]_i_ was increased in elderly ASM. To measure changes in [Ca^2+^]_i_ release following contractile agonist stimulation independent of influx in ASM histamine (10μM) was applied to Fura 2 AM loaded cells in 0 mM Ca^2+^ HBSS. Baseline in[Ca^2+^]_i_ was not changed (D). After histamine peak F) and amplitude (H) of [Ca^2+^]_i_ in elderly ASM. Data represents mean ± SEM from N of 6–7 patients. * indicates significant effect (p≤0.05).

To investigate whether age-related changes in [Ca^2+^]_i_ were specific to ACh or more generalized, histamine was used as an alternative agonist and experiments performed with 10 μM histamine in 2 mM vs. zero extracellular Ca^2+^. Histamine exposure resulted in a “biphasic” [Ca^2+^]_i_ response with an initial higher peak followed by a plateau before returning to baseline (Fig 6A): effects reduced in the absence of extracellular Ca^2+^ (Fig 6B). Aging did not change baseline Ca^2+^ in the presence or absence of extracellular Ca^2+^ (Fig 6C and 6D). Histamine increased peak (Fig 6E and 6F) and amplitude (Fig 6G and 6H) of [Ca^2+^]_i_ in elderly cells.

### SOCE and aging

**Fig 7:**
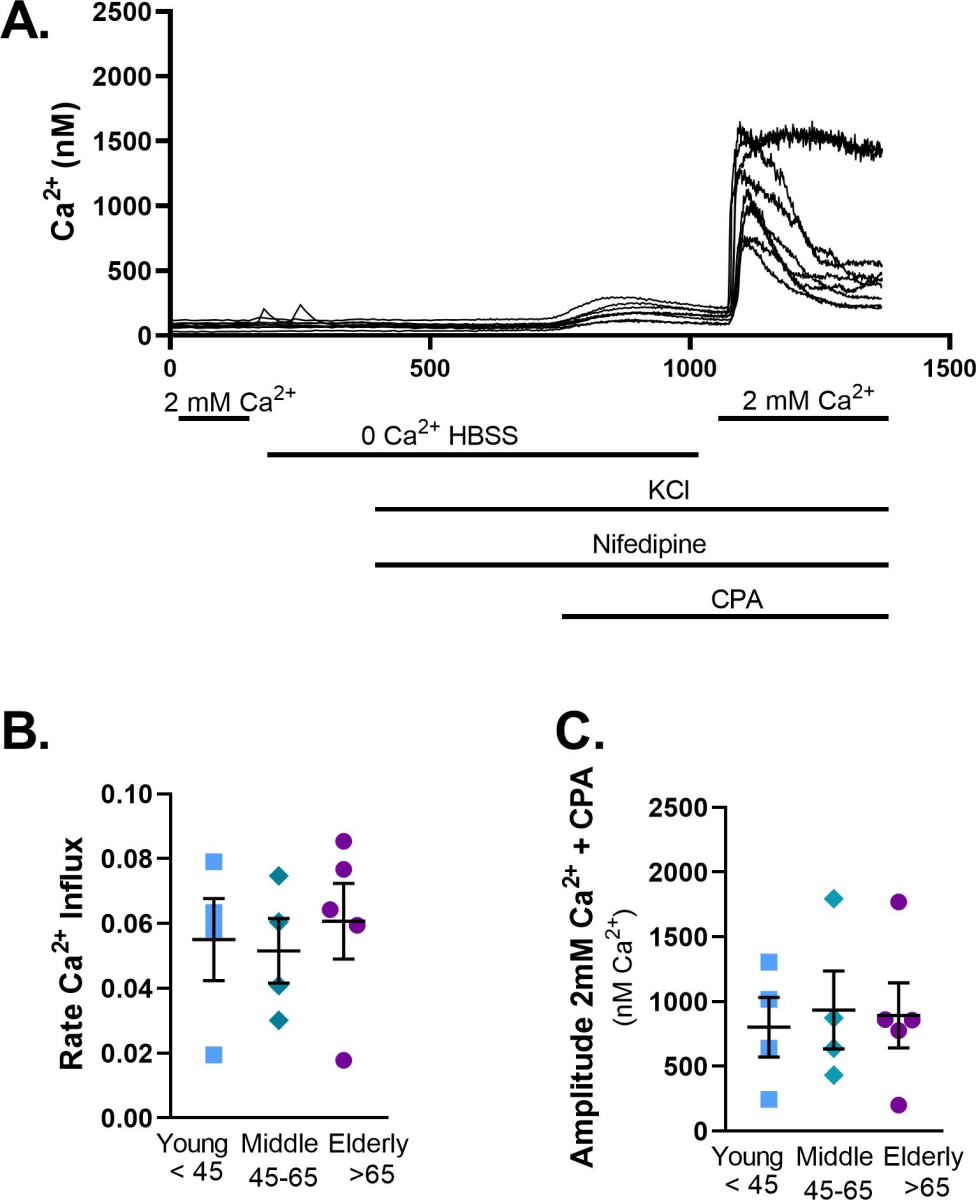
Store Operated Calcium Entry (SOCE) is not changed with age. Representative traces for SOCE in ASM (A). In the representative trace individual lines show the variations in cellular responses for a single well measured. The rate of Ca^2+^ influx was not changed with age (B). Similarly, the amplitude (C) of Ca^2+^ influx was not changed with age. Data represents mean ± SEM from N of 4–5 patients. * indicates significant effect (p≤0.05).

Age-related changes in SOCE was measured in young (representative examples shown in Fig 7A), middle age, and elderly ASM. There was no change in the rate of [Ca^2+^]_i_ increase (Fig 7B), or the amplitude (Fig 7C) of SOCE with age following reintroduction of Ca^2+^.

### Calcium regulatory proteins and aging

**Fig 8:**
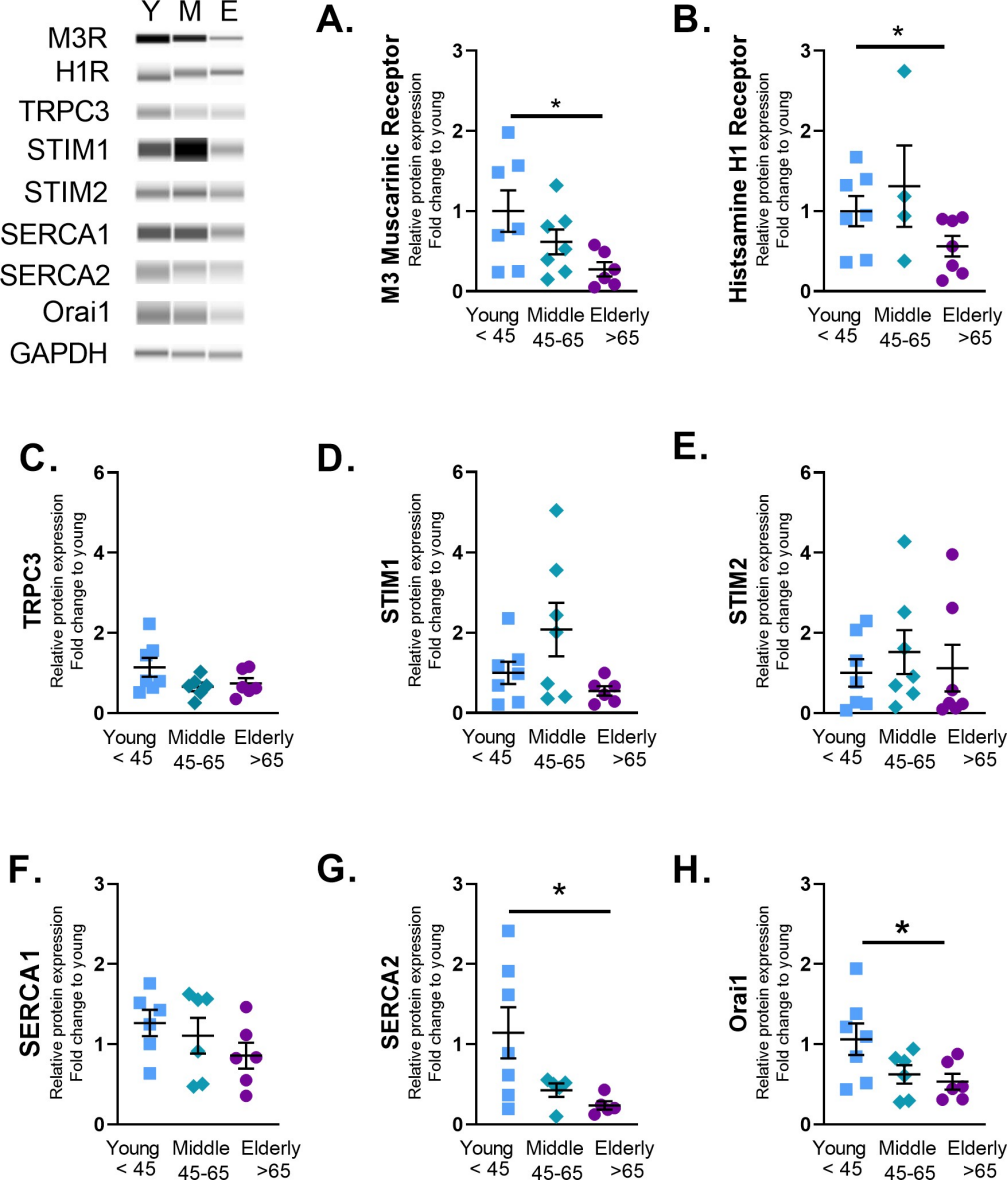
ASM Ca^2+^ signaling receptors expression decreased with age. Total protein was isolated from young <45, middle age 45–65, and elderly >65 ASM. Wes analysis was used to measure changes in protein expression. Graphs represent relative expression vs GAPDH. ASM isolated from elderly patients (>65) had significantly decreased M_3_ muscarinic receptor (A), histamine receptor (B), SERCA2 (G), and Orai1 (H) expression. Fold change was quantified using an average of young. Data shown as mean ± SEM from *n* = 4–7 patients. * indicates significant effect (p≤0.05).

To assess whether the enhanced [Ca^2+^]_i_ responses were due to altered expression of regulatory proteins, a variety of regulatory mechanisms were examined. Expression of M_3_ muscarinic receptor and histamine H1 receptor in fact decreased with age (Fig 8A and 8B) as do expression of SERCA 2 and Orai 1 (Fig 8G and 8H). Expression of TRPC3, STIM1 and STIM2, and SERCA1 did not show any significant changes with aging.

## Discussion

In this study, we report novel data on the changes that occur in ASM cells with normal aging. We chose 3 groups–young (<45 years of age), middle aged (45–65 years of age), and elderly (>65 years of age)–as defined by the WHO for comparison, towards demonstrating clinical significance of our studies. Our results indicate that aging results in increased cellular senescence of ASM. This increase in senescence is accompanied by a decrease in proliferation, but enhanced extracellular matrix deposition and SASP that can contribute to the thicker and potentially stiffer airways with aging. Interestingly, we also find that agonist-induced [Ca^2+^]_i_ responses increase in the elderly in spite of parallel data showing agonist receptor expression actually decreased in the elderly group, overall suggesting there may be a shift in sensitivity to [Ca^2+^]_i_ with aging.

Accumulation of senescent cells is known to contribute to the pathology of aging [23, 34]. Senescent cells release SASP that can lead to altered proliferation, upregulation of ER stress and altered unfolded protein response, mitochondrial dysfunction [20, 26, 50], fibrosis [30, 31, 33, 41] and inflammation [30–33]. In this regard, our data showing increased ASM senescence with aging become significant. ASM cells under baseline conditions show an upregulation of proteins associated with activation of senescent pathways such as p21, phospho γH2AX, and SA-β-gal. This increase in senescence marker expression is accompanied by changes in SASP release. Of note, changes in SASP elements are cell and context specific, and thus demonstration of aging-associated changes in human ASM *per se* is novel. ASM release of CCL2 decreases with age, while IL-8 increases with age. CCL2 is known to stimulate ASM proliferation [51], and thus our finding of decreased proliferation in aging ASM may be linked to CCL2.

Aging has significant effects on airway structure and function, presumably also reflecting recurrent cycles of environmental exposure, injury, inflammation, and repair [3]. At a macro level, aging results in an increase in lung stiffness [9, 11] and inflammation is assumed to be part of the aging process. While multiple cell types can contribute to aging associated changes in the airway, ASM is important given its role in contractility as well as in remodeling in the context of cell proliferation and fibrosis as occurs in diseases such as asthma [44, 45, 52], COPD [53], and even IPF [7]. However, there is very little known about ASM and ECM deposition with aging. Most data investigating aging and ECM deposition demonstrate a significant role of lung fibroblast during the aging process [31, 37, 54–56]. Here we show that aging increases ASM protein deposition of collagen III and fibronectin while MMP2 and MMP9 protein expression is decreased suggesting reduced ECM turnover. Furthermore, these data show that ASM is a major source of ECM deposition in the aging lung, thereby contributing to thicker more fibrotic airways.

Our observation of increased ECM (and decreased proliferation) with aging may seem to be in contrast to our findings of increased [Ca^2+^]_i_ responses of elderly ASM to bronchoconstrictor agonist. However, aging associated airway hyperreactivity in response to inhaled bronchoconstrictors such as histamine [57] and methacholine [58, 59], as well as loss of perception of bronchoconstriction [60], reduced bronchodilation [61], and increased airway tone [14, 62] have all been previously reported. What is less clear is whether such observations occur in the course of normal aging, or reflect an underlying reactive airway disease. Furthermore, the role of ASM per se in these clinical observations is not known.

To further investigate whether the observed increases in [Ca^2+^]_i_ responses in the elderly are due to enhanced Ca^2+^ influx versus enhanced SR Ca^2+^ release, we explored [Ca^2+^]_i_ responses in zero extracellular Ca^2+^. While the peak [Ca^2+^]_i_ responses were significantly decreased demonstrating a retained role for Ca^2+^ influx with aging, aging-associated increase in [Ca^2+^]_i_ was still observed suggesting alterations in intracellular Ca^2+^ regulatory pathways. Indeed, given aging-associated decrease in receptor expression, enhanced [Ca^2+^]_i_ responses were unlikely to be due this mechanism. Similarly, in spite of established roles for SOCE in ASM [63, 64], our findings of reduced STIM1 and Orai1 with aging suggest this mechanism is also unlikely to explain the higher [Ca^2+^]_i_ of aging ASM. In contrast, the decrease in SERCA expression could certainly contribute to retained increase in cytosolic Ca^2+^ with aging. In vascular smooth muscle cells, changes in [Ca^2+^]_i_ regulate adhesion to the ECM [65]. Furthermore, ECM stiffness acts as a switch that regulates whether force is transmitted through the ECM or through cell-cell connections [11]. Culturing ASM strips in the presence of collagen I, or fibronectin decreased maximal contraction [66]. Thus, increased [Ca^2+^]_i_ responses following agonist exposure may also be due to changes in ECM composition by ASM with age.

Senescence may also contribute to the observed changes in [Ca^2+^]_i_ responses with aging. Senescence is associated with increased ER stress and mitochondrial dysfunction [20, 22, 26, 50] uncoupling mitochondria and SR [67]. Furthermore, ATP production shifts from mitochondria to glycolysis with age [68]. This shift in the mechanism of ATP production is accompanied by an increase in Ca^2+^ stored in mitochondria [19, 21, 68]. In fibroblasts mitochondrial uncoupling leads to decreased [Ca^2+^]_i_ reuptake in senescent cells [20]. Thus, increased senescence in elderly ASM may result in increased Ca^2+^ mobilization from a combination of ER and mitochondrial stores and delayed reuptake in senescent cells due to decreased mitochondrial coupling. The contribution of such mechanisms in ASM with aging remain to be established.

Overall, these data demonstrate increased senescence with age in ASM cells. While proliferation decreases with age, ECM deposition and agonist-induced [Ca^2+^]_i_ responses are enhanced in the elderly. These findings are consistent with thickened and fibrotic airways in the aging lung. From a pathophysiological perspective, exacerbation of these aging-associated changes may explain the higher airway reactivity and remodeling of asthma with aging [3]. Accordingly, understanding ASM mechanisms in aging become important, and point to future research directions.

## Supporting information

S1 Table Gene expression for S16, p21, p16, and p53 were analyzed using qRT-PCR. The Ct value range for each age group are listed under the gene name.
(DOCX)

S1 Raw images (PDF)

## Abbreviations

ASM: Airway smooth muscle
ACh: Acetylcholine
β-gal: Beta galactosidase
Ca^2+^: Calcium
[Ca^2+^]_i_: Intracellular calcium concentration
CCL2: monocyte chemoattractant protein 1
ECM: Extracellular matrix
HBSS: Hank’s Balanced Salt Solution
IPF: Idiopathic Pulmonary Fibrosis
SASP: Senescence Associated Secretory Phenotype
SERCA: Sarcoplasmic reticulum Ca^2+^-ATPase
SR: Sarcoplasmic reticulum
STIM: Stromal interaction molecule
TRPC3: Transient receptor potential channel 3
