# Supplementary material for: Aging increases senescence, calcium signaling, and extracellular matrix deposition in human airway smooth muscle
Source: PLoS One. 2021 Jul 29;16(7):e0254710. doi: 10.1371/journal.pone.0254710 (PMC8321097; doi:10.1371/journal.pone.0254710)
Supplement: S1 Table — The Ct value range for each age group are listed under the gene name. (DOCX) [file pone.0254710.s001.docx]

S1 Table. qRT-PCR Ct values across age groups

|  | **qRT-PCR Ct Values** | | | |
| --- | --- | --- | --- | --- |
|  | S16 | P21 | P16 | P53 |
| Young | 17.1 - 18.0 | 17.9 - 19.4 | 23.6 - 25.1 | 23.0 - 24.5 |
| Middle Age | 17.7 - 20.9 | 17.9 - 21.3 | 23.4 - 27.9 | 23.1 - 26.5 |
| Elderly | 17.9 - 20.5 | 18.2 - 20.7 | 25.1 - 27.9 | 23.6 - 25.8 |
