# Supplementary material for: Aging increases senescence, calcium signaling, and extracellular matrix deposition in human airway smooth muscle
Source: PLoS One. 2021 Jul 29;16(7):e0254710. doi: 10.1371/journal.pone.0254710 (PMC8321097; doi:10.1371/journal.pone.0254710)
Supplement: S1 Raw images — (PDF) [file pone.0254710.s002.pdf]

S1_RAW_Images

Figure S1

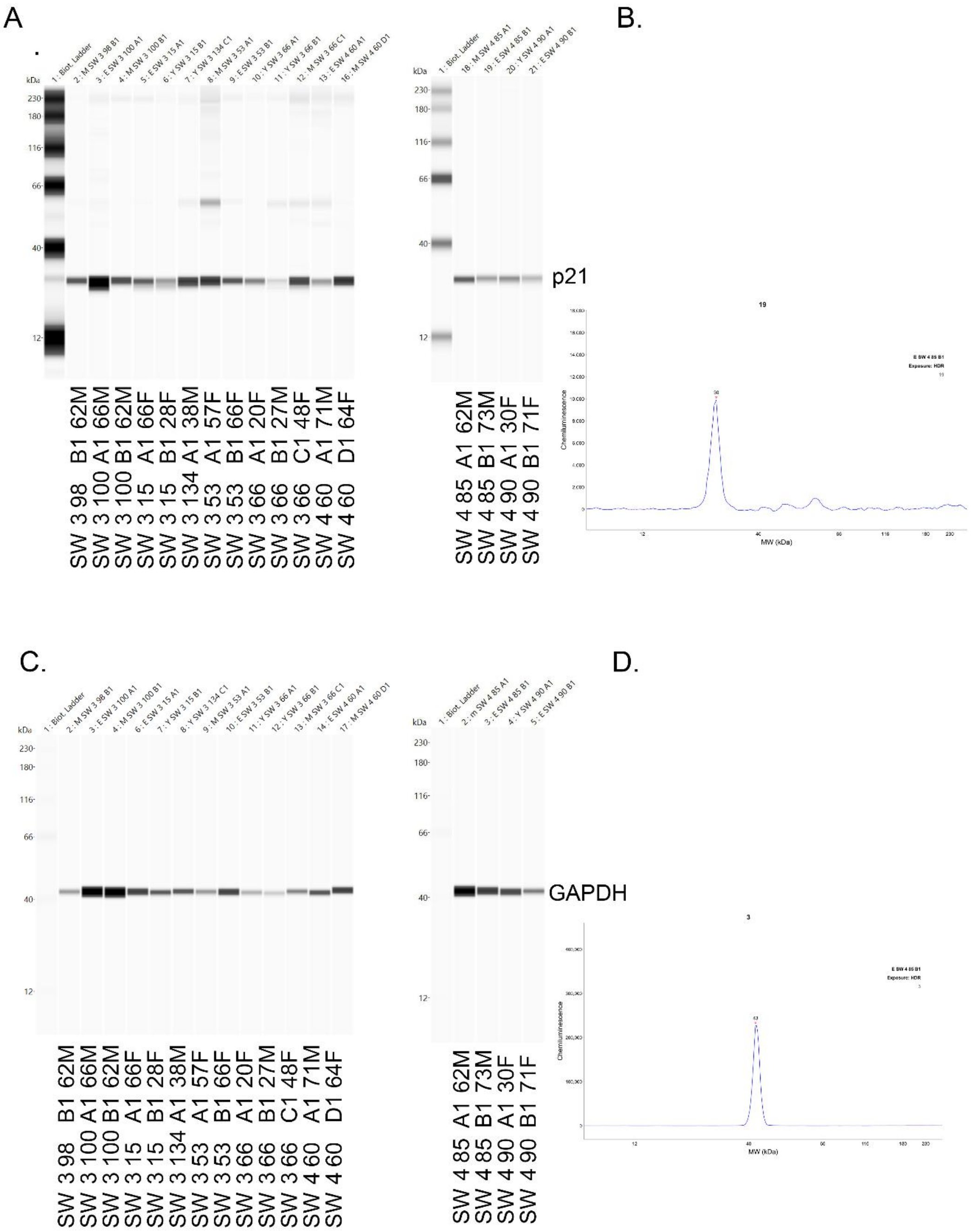

Figure S2

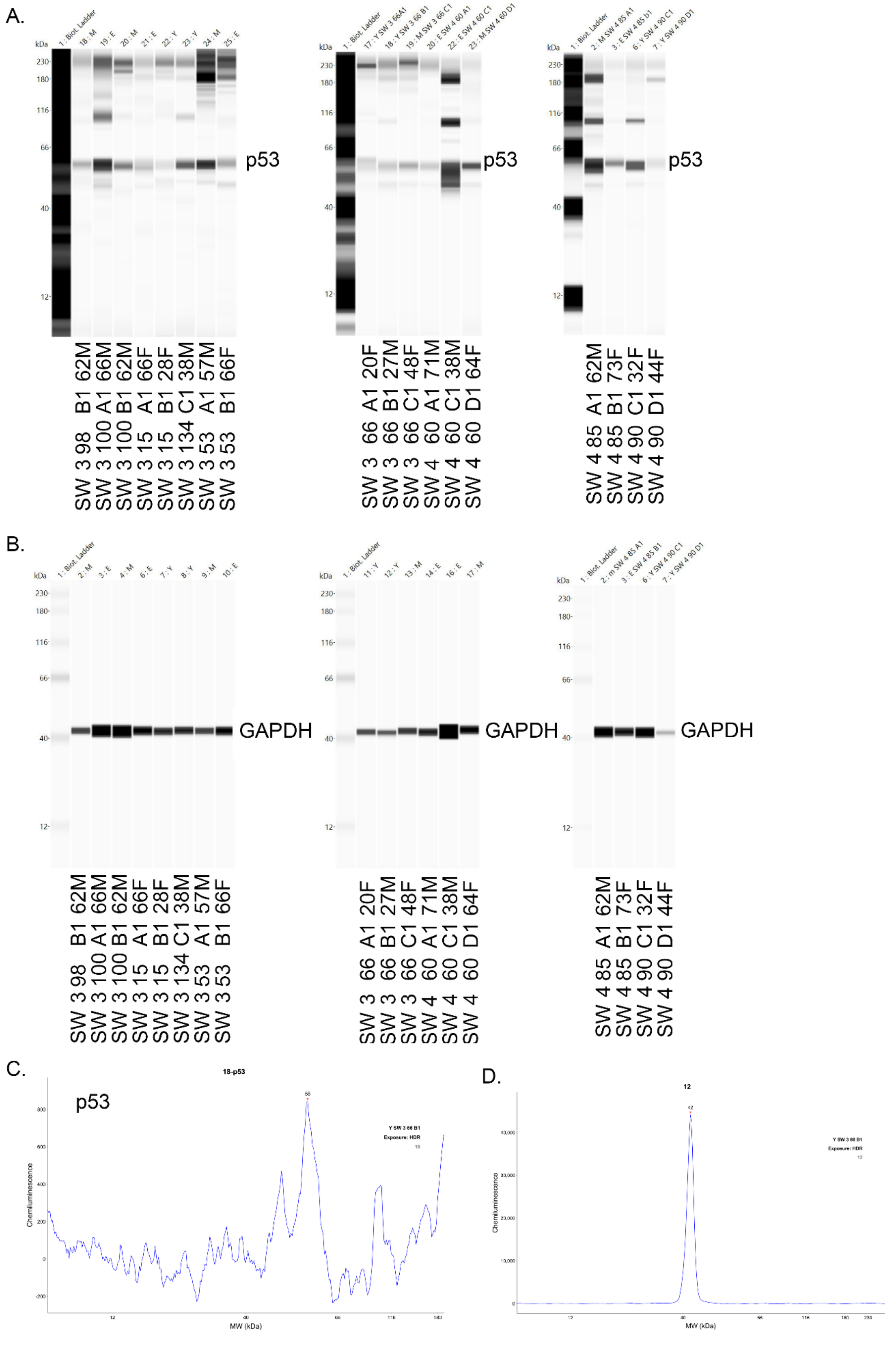

A.
p53
SW 3 98 B1 62M
SW 3 100 A1 66M
SW 3 100 B1 62M
SW 3 15 A1 66F
SW 3 15 B1 28F
SW 3 134 C1 38M
SW 3 53 A1 57M
SW 3 53 B1 66F
SW 3 66 A1 20F
SW 3 66 B1 27M
SW 3 66 C1 48F
SW 4 60 A1 71M
SW 4 60 C1 38M
SW 4 60 D1 64F
SW 4 85 A1 62M
SW 4 85 B1 73F
SW 4 90 C1 32F
SW 4 90 D1 44F
B.
GAPDH
C.
p53
Chemiluminescence
MW (kDa)
D.
Chemiluminescence
MW (kDa)

Figure S3

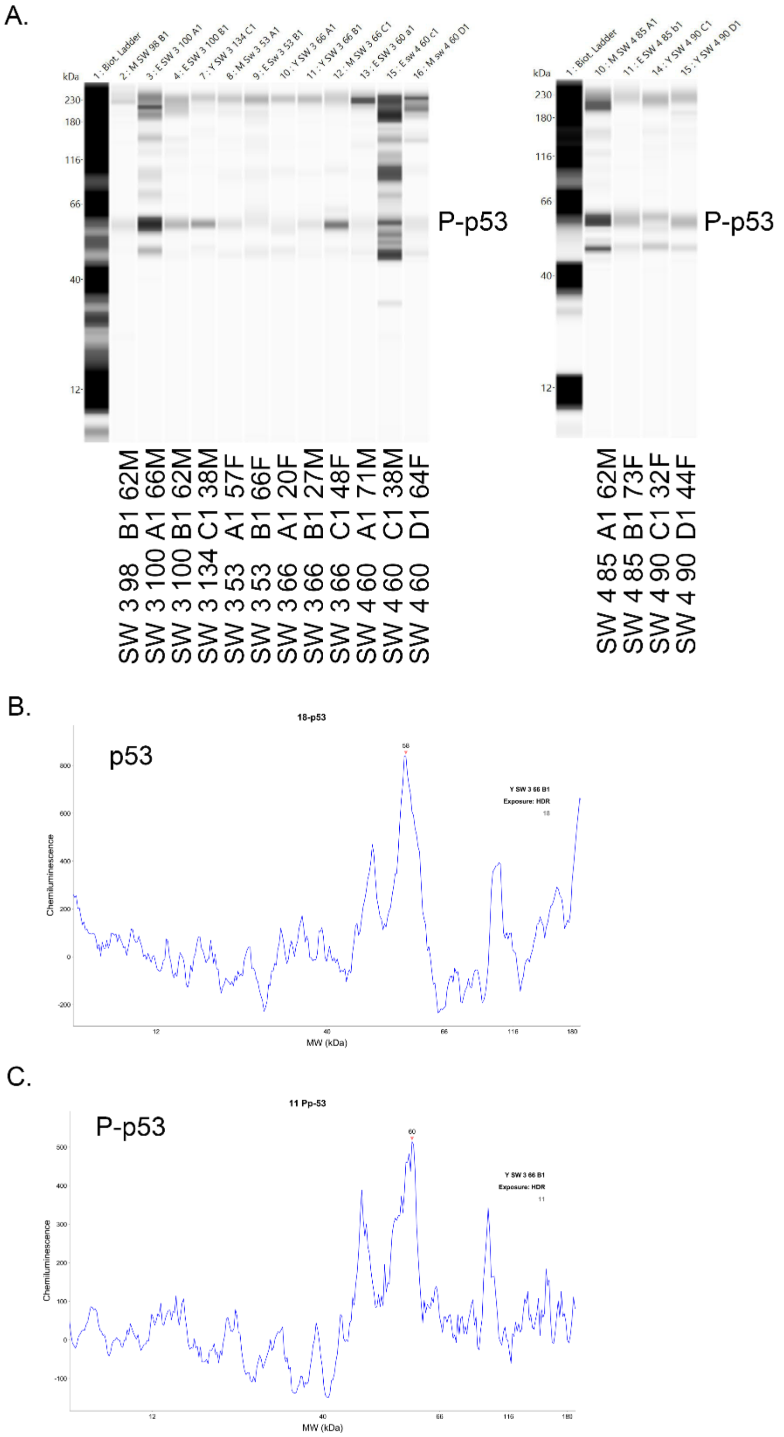

A.
kDa
230
180
116
66
40
12
P-p53
SW 3 98 B1 62M
SW 3 100 A1 66M
SW 3 100 B1 62M
SW 3 134 C1 38M
SW 3 53 A1 57F
SW 3 53 B1 66F
SW 3 66 A1 20F
SW 3 66 B1 27M
SW 3 66 C1 48F
SW 4 60 A1 71M
SW 4 60 C1 38M
SW 4 60 D1 64F
SW 4 85 A1 62M
SW 4 85 B1 73F
SW 4 90 C1 32F
SW 4 90 D1 44F
B.
18-p53
p53
Y SW 3 66 B1
Exposure: HDR
Chemiluminescence
MW (kDa)
C.
11 Pp-53
P-p53
Y SW 3 66 B1
Exposure: HDR
Chemiluminescence
MW (kDa)

Figure S4

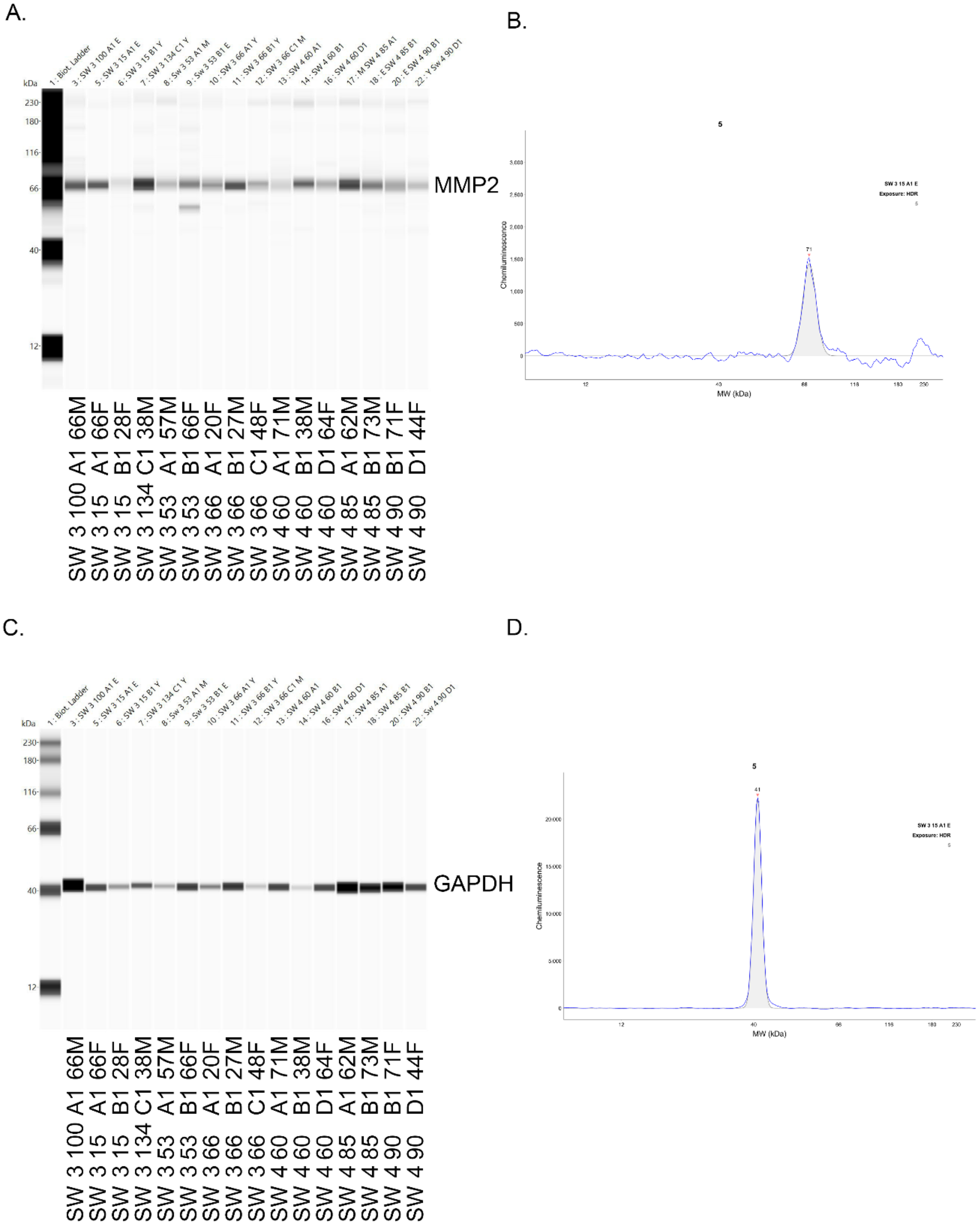
A.
B.
C.
D.
kDa
MMP2
GAPDH
SW 3 100 A1 66M
SW 3 15 A1 66F
SW 3 15 B1 28F
SW 3 134 C1 38M
SW 3 53 A1 57M
SW 3 53 B1 66F
SW 3 66 A1 20F
SW 3 66 B1 27M
SW 3 66 C1 48F
SW 4 60 A1 71M
SW 4 60 B1 38M
SW 4 60 D1 64F
SW 4 85 A1 62M
SW 4 85 B1 73M
SW 4 90 B1 71F
SW 4 90 D1 44F
Chemiluminescence
MW (kDa)
SW 3 15 A1 E
Exposure: HDR

Figure S5

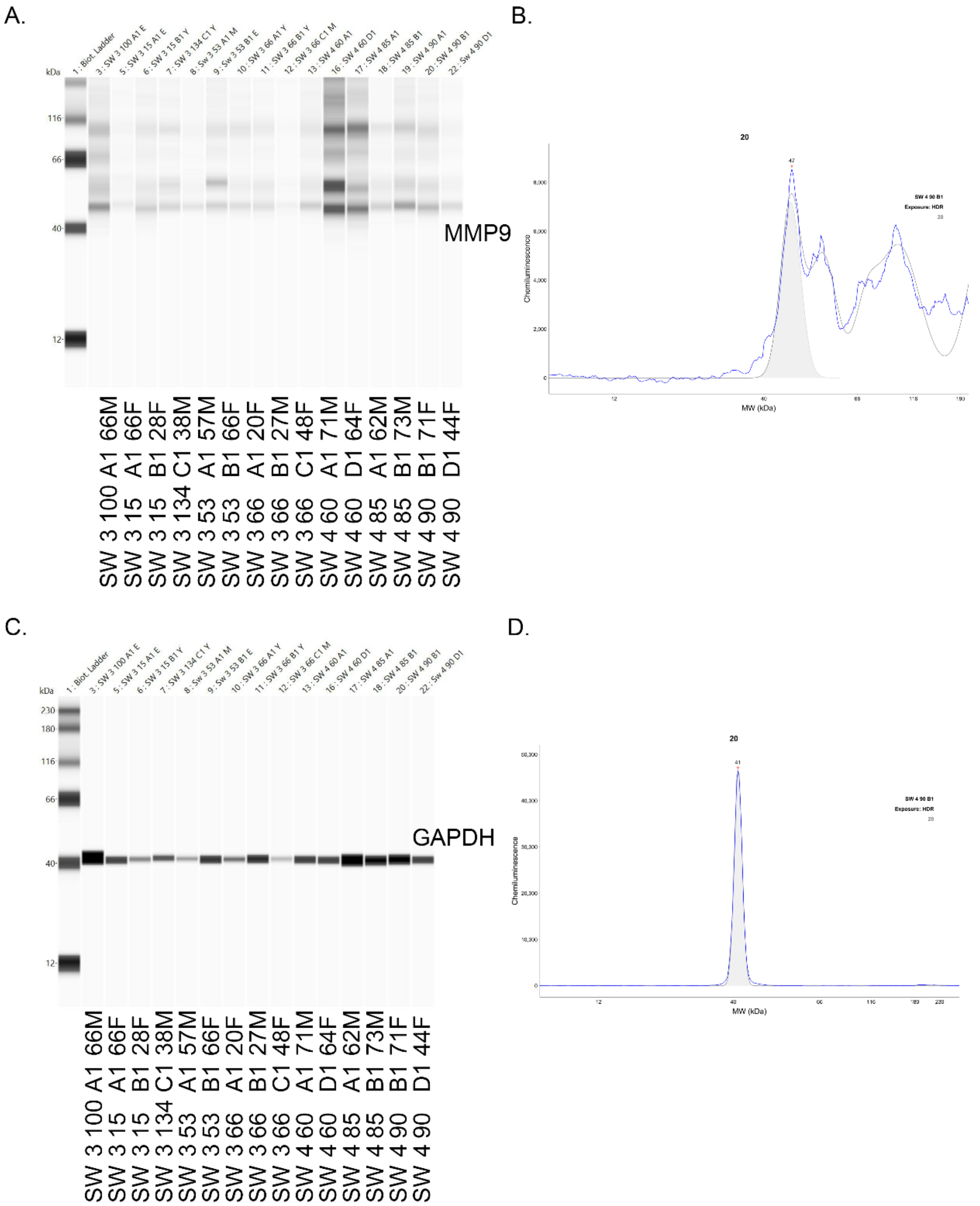


Figure S6

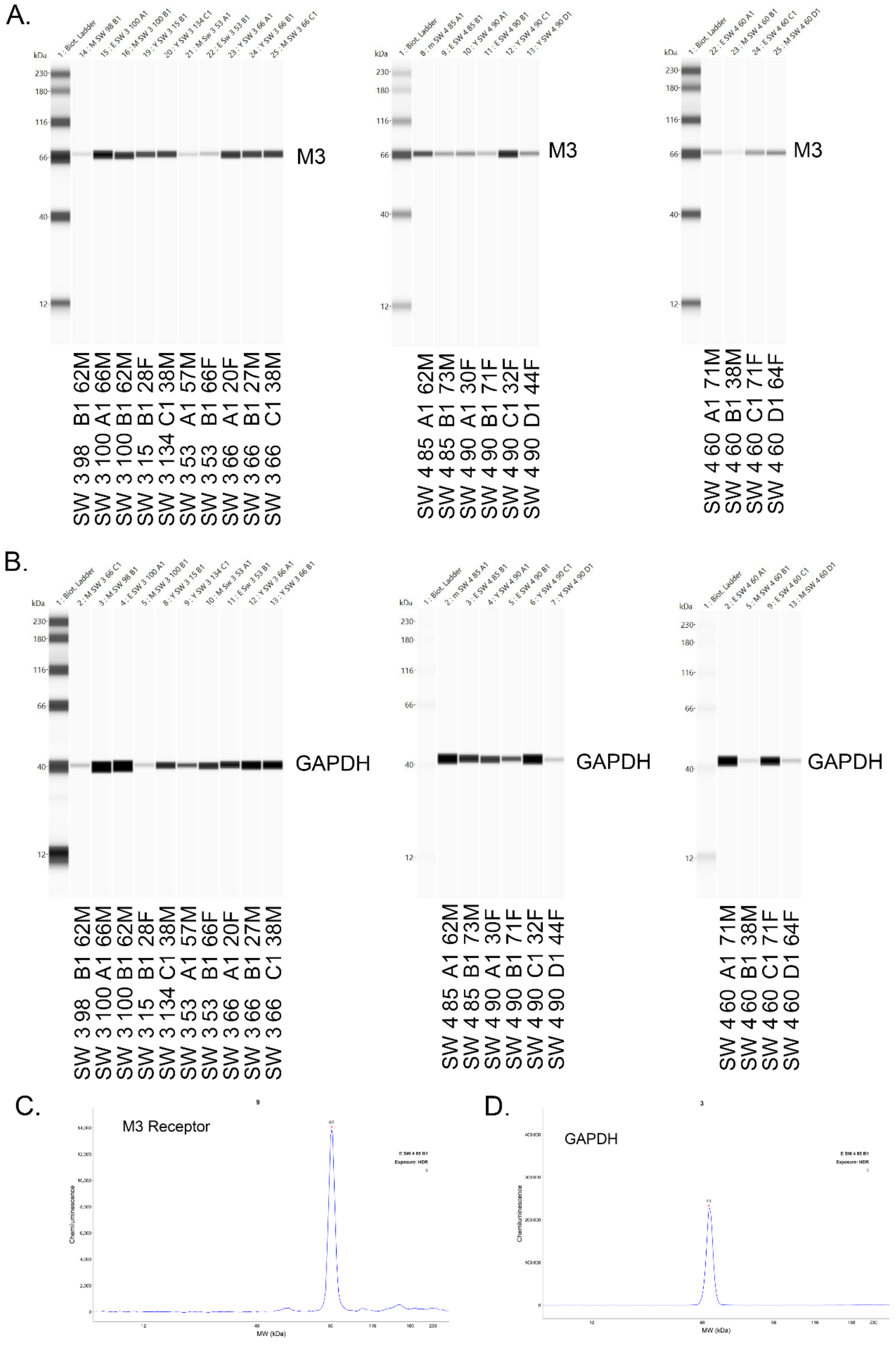


Figure S7

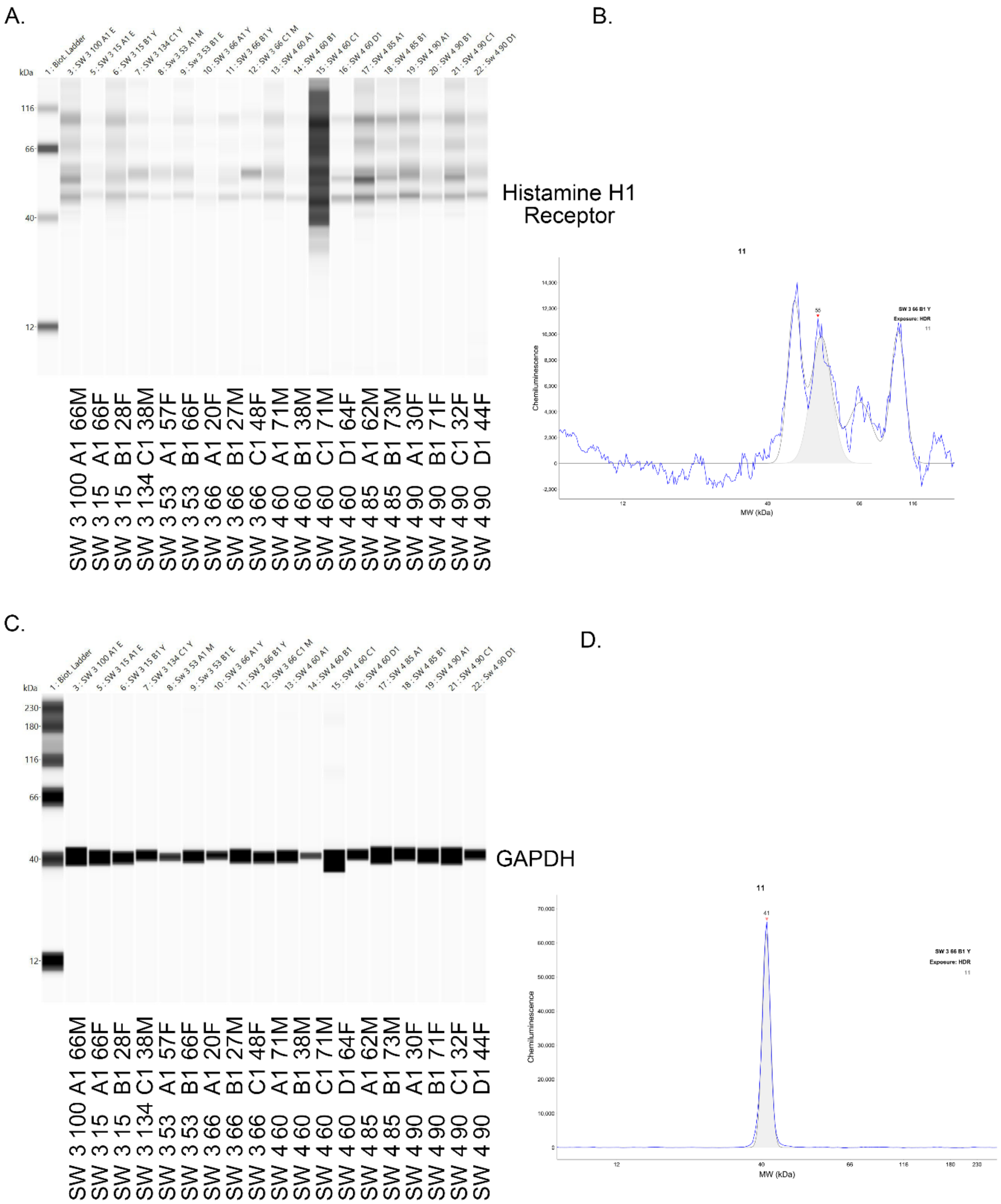
A.
B.
C.
D.
Histamine H1 Receptor
GAPDH
kDa
SW 3 100 A1 66M
SW 3 15 A1 66F
SW 3 15 B1 28F
SW 3 134 C1 38M
SW 3 53 A1 57F
SW 3 53 B1 66F
SW 3 66 A1 20F
SW 3 66 B1 27M
SW 3 66 C1 48F
SW 4 60 A1 71M
SW 4 60 B1 38M
SW 4 60 C1 71M
SW 4 60 D1 64F
SW 4 85 A1 62M
SW 4 85 B1 73M
SW 4 90 A1 30F
SW 4 90 B1 71F
SW 4 90 C1 32F
SW 4 90 D1 44F
Chemiluminescence
MW (kDa)
SW 3 66 B1 Y
Exposure: HDR

Figure S8

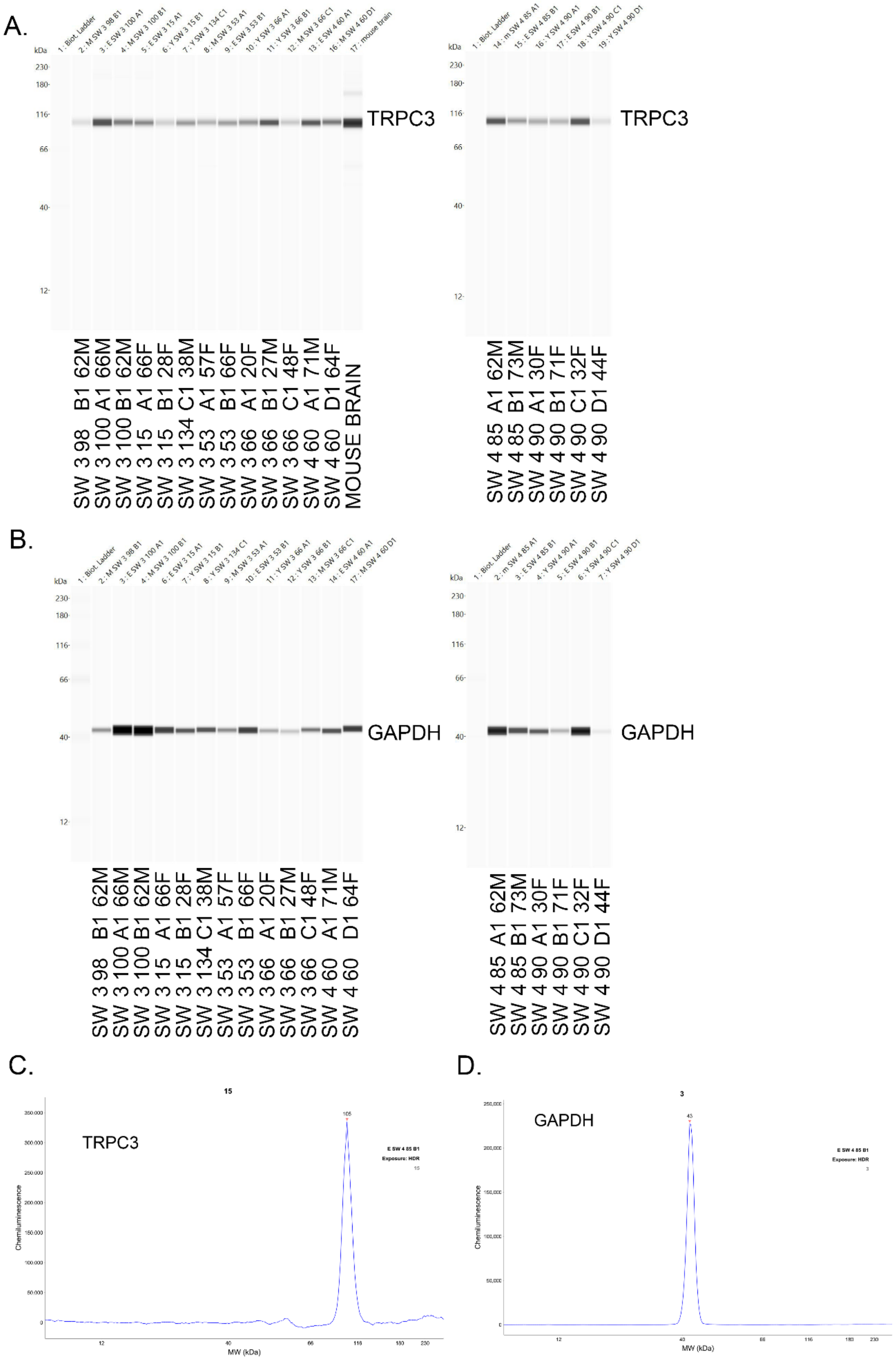
A.
TRPC3
TRPC3
SW 3 98 B1 62M
SW 3 100 A1 66M
SW 3 100 B1 62M
SW 3 15 A1 66F
SW 3 15 B1 28F
SW 3 134 C1 38M
SW 3 53 A1 57F
SW 3 53 B1 66F
SW 3 66 A1 20F
SW 3 66 B1 27M
SW 3 66 C1 48F
SW 4 60 A1 71M
SW 4 60 D1 64F
MOUSE BRAIN
SW 4 85 A1 62M
SW 4 85 B1 73M
SW 4 90 A1 30F
SW 4 90 B1 71F
SW 4 90 C1 32F
SW 4 90 D1 44F
B.
GAPDH
GAPDH
C.
TRPC3
Chemiluminescence
MW (kDa)
D.
GAPDH
Chemiluminescence
MW (kDa)

Figure S9

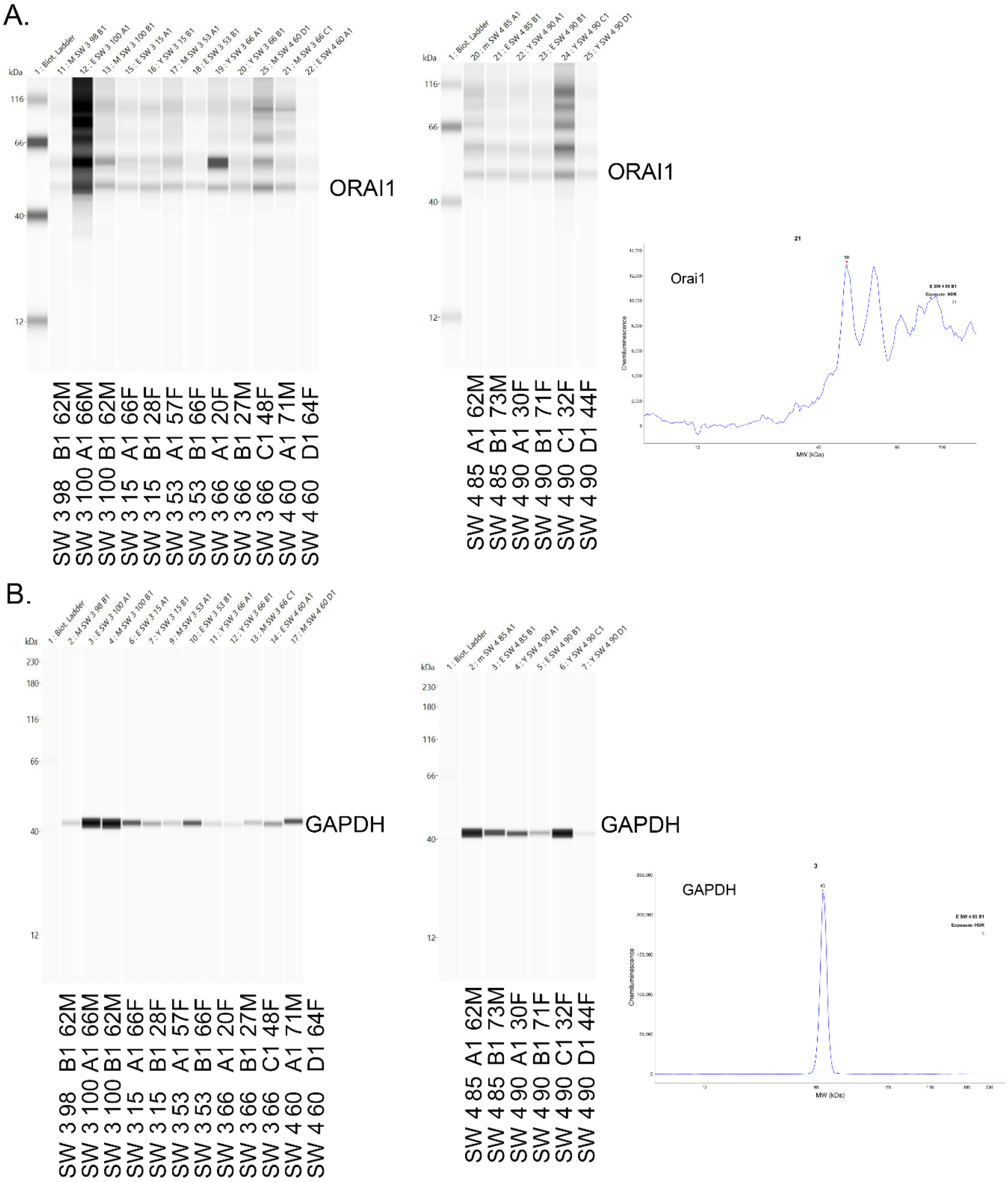
A.
ORAI1
ORAI1
Orai1
kDa
116
66
40
12
SW 3 98 B1 62M
SW 3 100 A1 66M
SW 3 100 B1 62M
SW 3 15 A1 66F
SW 3 15 B1 28F
SW 3 53 A1 57F
SW 3 53 B1 66F
SW 3 66 A1 20F
SW 3 66 B1 27M
SW 3 66 C1 48F
SW 4 60 A1 71M
SW 4 60 D1 64F
SW 4 85 A1 62M
SW 4 85 B1 73M
SW 4 90 A1 30F
SW 4 90 B1 71F
SW 4 90 C1 32F
SW 4 90 D1 44F
B.
GAPDH
GAPDH
GAPDH
kDa
230
180
116
66
40
12
SW 3 98 B1 62M
SW 3 100 A1 66M
SW 3 100 B1 62M
SW 3 15 A1 66F
SW 3 15 B1 28F
SW 3 53 A1 57F
SW 3 53 B1 66F
SW 3 66 A1 20F
SW 3 66 B1 27M
SW 3 66 C1 48F
SW 4 60 A1 71M
SW 4 60 D1 64F
SW 4 85 A1 62M
SW 4 85 B1 73M
SW 4 90 A1 30F
SW 4 90 B1 71F
SW 4 90 C1 32F
SW 4 90 D1 44F

Figure S10

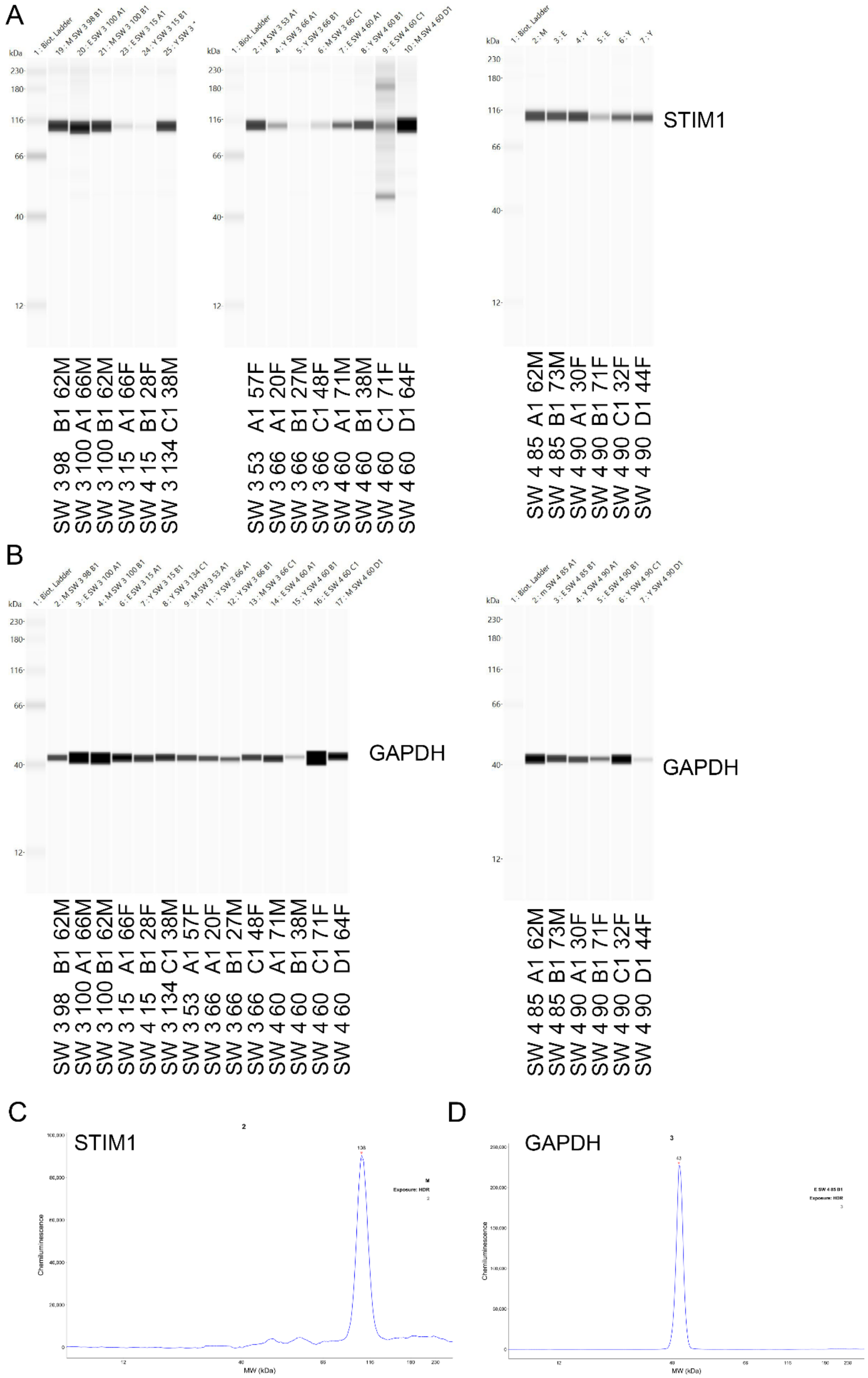
A
STIM1
SW 3 98 B1 62M
SW 3 100 A1 66M
SW 3 100 B1 62M
SW 3 15 A1 66F
SW 4 15 B1 28F
SW 3 134 C1 38M
SW 3 53 A1 57F
SW 3 66 A1 20F
SW 3 66 B1 27M
SW 3 66 C1 48F
SW 4 60 A1 71M
SW 4 60 B1 38M
SW 4 60 C1 71F
SW 4 60 D1 64F
SW 4 85 A1 62M
SW 4 85 B1 73M
SW 4 90 A1 30F
SW 4 90 B1 71F
SW 4 90 C1 32F
SW 4 90 D1 44F
B
GAPDH
C
STIM1
Chemiluminescence
MW (kDa)
D
GAPDH

Figure S11

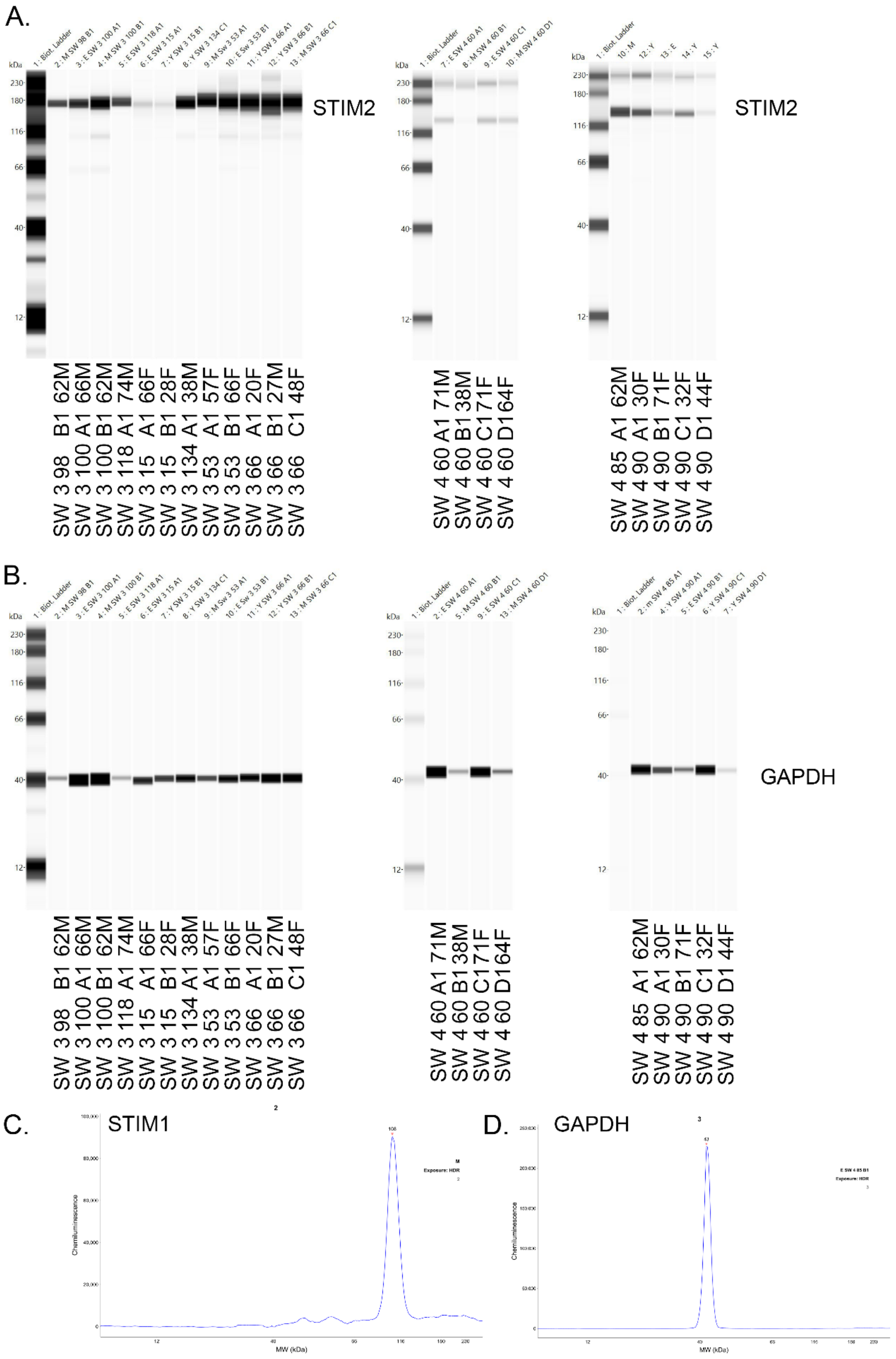
A.
STIM2
STIM2
SW 3 98 B1 62M
SW 3 100 A1 66M
SW 3 100 B1 62M
SW 3 118 A1 74M
SW 3 15 A1 66F
SW 3 15 B1 28F
SW 3 134 A1 38M
SW 3 53 A1 57F
SW 3 53 B1 66F
SW 3 66 A1 20F
SW 3 66 B1 27M
SW 3 66 C1 48F
SW 4 60 A1 71M
SW 4 60 B1 38M
SW 4 60 C171F
SW 4 60 D164F
SW 4 85 A1 62M
SW 4 90 A1 30F
SW 4 90 B1 71F
SW 4 90 C1 32F
SW 4 90 D1 44F
B.
GAPDH
SW 3 98 B1 62M
SW 3 100 A1 66M
SW 3 100 B1 62M
SW 3 118 A1 74M
SW 3 15 A1 66F
SW 3 15 B1 28F
SW 3 134 A1 38M
SW 3 53 A1 57F
SW 3 53 B1 66F
SW 3 66 A1 20F
SW 3 66 B1 27M
SW 3 66 C1 48F
SW 4 60 A1 71M
SW 4 60 B1 38M
SW 4 60 C171F
SW 4 60 D164F
SW 4 85 A1 62M
SW 4 90 A1 30F
SW 4 90 B1 71F
SW 4 90 C1 32F
SW 4 90 D1 44F
C.
STIM1
D.
GAPDH

Figure S12

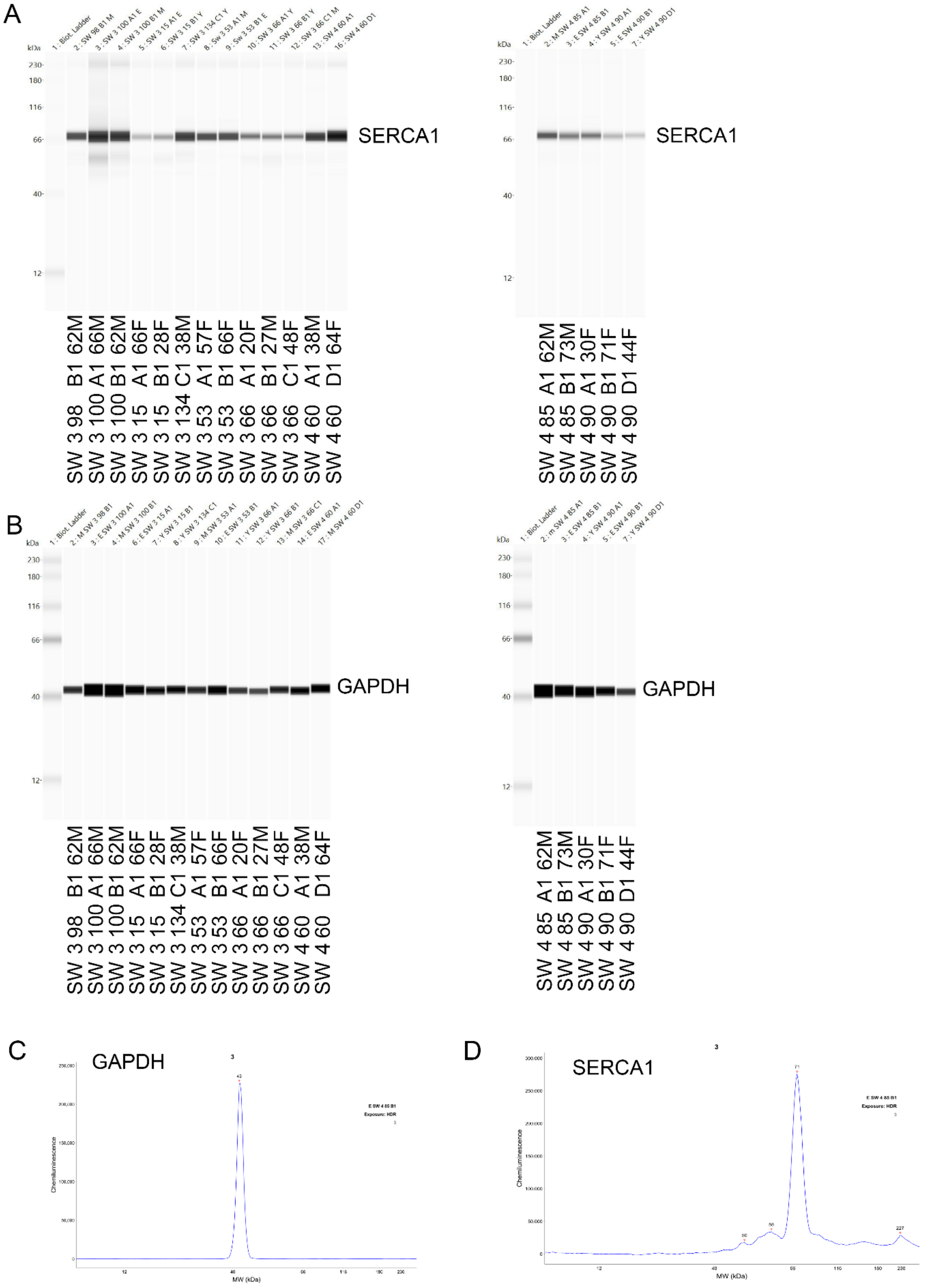
A
SERCA1
SERCA1
SW 3 98 B1 62M
SW 3 100 A1 66M
SW 3 100 B1 62M
SW 3 15 A1 66F
SW 3 15 B1 28F
SW 3 134 C1 38M
SW 3 53 A1 57F
SW 3 53 B1 66F
SW 3 66 A1 20F
SW 3 66 B1 27M
SW 3 66 C1 48F
SW 4 60 A1 38M
SW 4 60 D1 64F
SW 4 85 A1 62M
SW 4 85 B1 73M
SW 4 90 A1 30F
SW 4 90 B1 71F
SW 4 90 D1 44F
B
GAPDH
GAPDH
C
GAPDH
D
SERCA1
Chemiluminescence
MW (kDa)

Figure S13

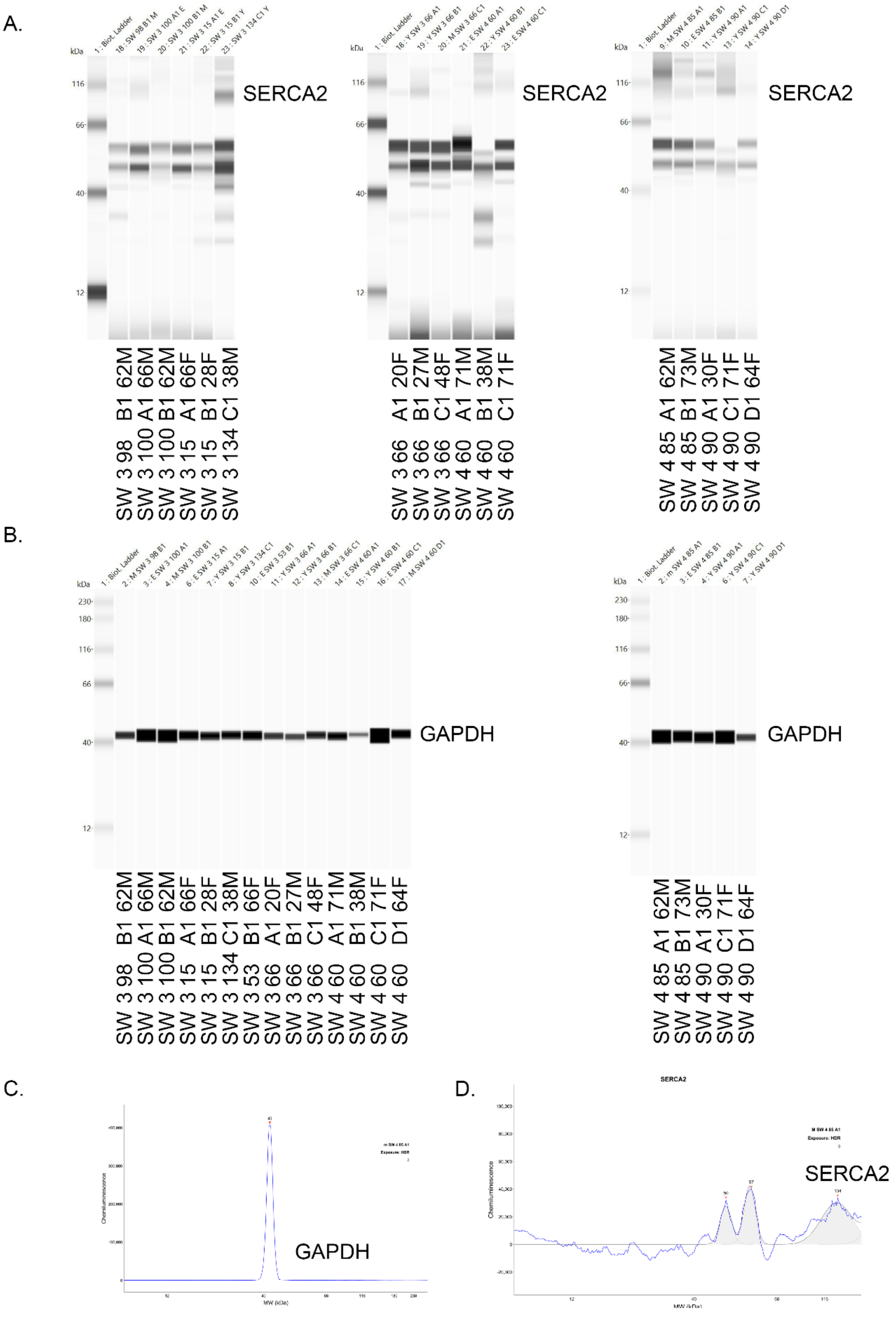
A.
SERCA2
SERCA2
SERCA2
SW 3 98 B1 62M
SW 3 100 A1 66M
SW 3 100 B1 62M
SW 3 15 A1 66F
SW 3 15 B1 28F
SW 3 134 C1 38M
SW 3 66 A1 20F
SW 3 66 B1 27M
SW 3 66 C1 48F
SW 4 60 A1 71M
SW 4 60 B1 38M
SW 4 60 C1 71F
SW 4 85 A1 62M
SW 4 85 B1 73M
SW 4 90 A1 30F
SW 4 90 C1 71F
SW 4 90 D1 64F
B.
GAPDH
GAPDH
SW 3 98 B1 62M
SW 3 100 A1 66M
SW 3 100 B1 62M
SW 3 15 A1 66F
SW 3 15 B1 28F
SW 3 134 C1 38M
SW 3 53 B1 66F
SW 3 66 A1 20F
SW 3 66 B1 27M
SW 3 66 C1 48F
SW 4 60 A1 71M
SW 4 60 B1 38M
SW 4 60 C1 71F
SW 4 60 D1 64F
SW 4 85 A1 62M
SW 4 85 B1 73M
SW 4 90 A1 30F
SW 4 90 C1 71F
SW 4 90 D1 64F
C.
GAPDH
D.
SERCA2
SERCA2

**Figure S1**. P21 western blot generated using WES a capillary western system. The wes system creates a figure that looks like a traditional western blot (A). Representative trace for p21 (B) and GAPDH (D). p21 protein expression was quantified by measuring the area under the curve for p21(B) normalized to the area under the curve for GAPDH (D). The sample name and age has been annotated below each lane.

**Figure S2**. P53 western blot generated using WES a capillary western system. The wes system creates a figure that looks like a traditional western blot (A). Representative trace for p53 (C) and GAPDH (D). p53 protein expression was quantified by measuring the area under the curve for p53(C) normalized to the area under the curve for GAPDH (D). The sample name and age has been annotated below each lane.

**Figure S3**. Western blot for phospho p53 (P-p53) (A). A representative trace for p53 (B) and Pp53 (C). The sample age and name are annotated below each lane. P-p53 was quantified to P53 data shown in Figure S2. The age and sample ID has been annotated below each lane.

**Figure S4.** Western blot  for MMP2 was generated via WES Simple Western. GAPDH used to normalize MMP2 gene expression is shown in (C). MMP2 expression was quantified by measuring the area under the curve for MMP2 (B) vs area under the curve for GAPDH (D). The age and sample ID has been annotated below each lane.

**Figure S5.** Western blot  for MMP9 was generated via WES Simple Western. GAPDH used to normalize MMP9 gene expression is shown in (C). MMP9 expression was quantified by measuring the area under the curve for MMP9 (B) vs area under the curve for GAPDH (D). The age and sample ID has been annotated below each lane.

**Figure S6.** Western blot  for M3 muscarinic receptor was generated via WES Simple Western. GAPDH used to normalize M3 muscarinic receptor expression is shown in (B). M3 muscarinic receptor expression was quantified by measuring the area under the curve for M3 (C) vs area under the curve for GAPDH (D). The age and sample ID has been annotated below each lane.

**Figure S7** Western blot for histamine H1 receptor generated via WES simple western (A). H1R expression was normalized to  GAPDH (C). Representative trace showing how protein expression was quantified by area under the curve of histamine H1R (B) normalized to GAPDH (D). The age and sample ID is annotated below each lane.

**Figure S8.** TRPC3 western blot generated using WES a capillary western system. The wes system creates a figure that looks like a traditional western blot (A). Representative trace for TRPC3 (C) and GAPDH (D). TRPC3 protein expression was quantified by measuring the area under the curve for TRPC3(C) normalized to the area under the curve for GAPDH (D). The sample name and age has been annotated below each lane.

**Figure S9.** Western blot for Orai1 was generated via WES Simple Western. GAPDH used to normalize Orai1 expression is shown in (C). Orai1 protein expression was quantified by measuring the area under

the curve for Orai1 (B) vs area under the curve for GAPDH (D). The age and sample ID has been annotated below each lane.

**Figure S10**. STIM1 western blot generated using WES a capillary western system (A). Representative trace for STIM1 (C) and GAPDH (D). STIM1 protein expression was quantified by measuring the area under the curve for STIM1(C) normalized to the area under the curve for GAPDH (D). The sample name and age has been annotated below each lane.

**Figure S11**. STIM2 western blot generated using WES a capillary western system(A). Representative trace for STIM2 (C) and GAPDH (D). STIM2 protein expression was quantified by measuring the area under the curve for STIM2(C) normalized to the area under the curve for GAPDH (D). The sample name and age has been annotated below each lane.

**Figure S12.** SERCA1 western blot generated using WES a capillary western system. The wes system creates a figure that looks like a traditional western blot (A). Representative trace for SERCA1 (C) and GAPDH (D). SERCA1 protein expression was quantified by measuring the area under the curve for SERCA1 (C) normalized to the area under the curve for GAPDH (D). The sample name and age has been annotated below each lane.

**Figure S13**. SERCA2 western blot generated using WES a capillary western system. The wes system creates a figure that looks like a traditional western blot (A). Representative trace for SERCA2 (C) and GAPDH (D). SERCA2 protein expression was quantified by measuring the area under the curve for SERCA2 (C) normalized to the area under the curve for GAPDH (D). The sample name and age has been annotated below each lane.
